# Remote Modulation of Single‐Atom Catalyst Boosts High‐Valent Cobalt–Oxo Species Generation for Water Purification and Detoxification

**DOI:** 10.1002/advs.202512498

**Published:** 2025-12-12

**Authors:** Wen‐Min Wang, Zheng‐Wei Yang, De‐Xiu Wu, Wen‐Long Wang, Qian‐Yuan Wu

**Affiliations:** ^1^ Shenzhen Key Laboratory of Ecological Remediation and Carbon Sequestration Environmental Protection Key Laboratory of Microorganism Application and Risk Control Institute of Environment and Ecology Shenzhen International Graduate School Tsinghua University Shenzhen 518055 P. R. China; ^2^ Key Laboratory of Microorganism Application and Risk Control of Shenzhen Guangdong Provincial Engineering Research Center for Urban Water Recycling and Environmental Safety Institute of Environment and Ecology Shenzhen International Graduate School Tsinghua University Shenzhen 518055 P. R. China

**Keywords:** high‐valent cobalt‐oxo species, peroxymonosulfate activation, remote modulation, single‐atom catalysts, toxicity

## Abstract

With a high redox potential and long half‐life, high‐valent cobalt–oxo species (Co(IV)═O) hold promise for water purification by eliminating persistent contaminants. However, the inefficient and unsustainable generation of Co(IV)═O limits its practical application. In this work, a phosphorus (P)‐doped cobalt single‐atom catalyst (Co─N_6_/C─P) is developed, where P‐substituted nitrogen (N) atoms are coordinated to the cobalt site at meta‐positions. This remote modulation reduces the charge density of the cobalt site and positively shifts the d‐band center of the cobalt atom, thereby lowering the energy barrier for Co(IV)═O generation. The P‐doping increases the turnover frequency of the cobalt center by 3.5 times and the steady‐state concentration of Co(IV)═O by 2.7 times. The (Co─N_6_/C─P)/peroxymonosulfate (PMS) system exhibits a pollutant degradation kinetic constant three times higher than that of Co─N_6_/C, surpassing most reported single‐atom catalytic PMS systems. A continuous‐flow reactor based on Co─N_6_/C─P achieves over 87% contaminant removal after 24 h of operation. The treated real wastewater exhibits exceptionally low cytotoxicity (2.96 mg‐phenol L^−1^) and genotoxicity (0.08 µg‐4‐NQO L^−1^) to mammalian cells, enhancing water safety. This study presents a reliable approach for the removal of persistent contaminants and the reduction of toxicity through efficient Co(IV)═O generation enabled by a remote modulation strategy.

## Graphical abstract

This study develops a phosphorus‐doped cobalt single‐atom catalyst that enhances high‐valent cobalt–oxo species generation for water purification. This atomic‐level tuning boosts the catalyst's activity by 3.5 times, enabling efficient pollutant degradation. A continuous‐flow reactor achieves high, long‐term removal efficiency, producing water with low toxicity, offering a promising approach for environmental purification.

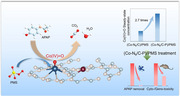

## 1 Introduction

Pharmaceutical and personal care products pose potential threats to both aquatic organisms and human health due to their recalcitrant properties and ecological toxicity.^[^ ^1^, ^2^ ^]^ Advanced oxidation processes (AOPs) based on peroxymonosulfate (PMS) have demonstrated excellent efficacy in removing recalcitrant pollutants for water decontamination.^[^ ^3^, ^4^ ^]^ However, existing heterogeneous catalysts face fundamental challenges, including poor stability, low atomic utilization efficiency, and slow activation kinetics.^[^ ^5^, ^6^ ^]^ Additionally, the surface chemistry of these catalysts is complex and difficult to control precisely, which poses challenges in identifying active sites and analyzing catalytic mechanisms.^[^ ^7^ ^]^

Single‐atom catalysts (SACs) are one of the most promising catalysts for PMS‐AOPs due to their utmost atomic utilization efficiency, significantly enhanced catalytic properties, and well‐defined structures.^[^ ^8^, ^9^, ^10^ ^]^ Cobalt (Co)‐based SACs, in particular, have been reported as the most active catalysts for this application.^[^ ^11^, ^12^ ^]^ For instance, Co SACs with N‐coordination demonstrate catalytic activities that are 2 to 4 orders of magnitude higher than those of Co^2+^ and Co_3_O_4_ in PMS activation and the elimination of the micropollutant bisphenol A.^[^ ^13^ ^]^ SACs activate PMS to generate reactive oxidative species, such as sulfate and hydroxyl radicals, singlet oxygen, and high‐valent metal‐oxo species, which are essential for pollutant degradation.^[^ ^14^, ^15^ ^]^ Among these, high‐valent metal–oxo species exhibit high redox potentials, long half‐lives, and strong resistance to complex water matrices, making them particularly suitable for the selective removal of recalcitrant pollutants under challenging water quality conditions.^[^ ^16^, ^17^, ^18^ ^]^ The half‐lives of high‐valent metal–oxo species (7–10 s) are orders of magnitude longer than those of other reactive species (10^−3^ µs for ^•^OH, 30–40 µs for SO_4_^•−^, and 2 µs for ^1^O_2_), enabling sufficient interaction with contaminants in aqueous environments.^[^ ^11^, ^19^, ^20^, ^21^ ^]^ Additionally, high‐valent metal–oxo species can selectively react with electron‐rich micropollutants at moderate rates. Consequently, high‐valent metal–oxo species hold great promise in the field of advanced water purification. However, effective technologies for the efficient and selective generation of high‐valent metal–oxo species remain underdeveloped, as ^•^OH and SO_4_^•−^ are generally considered the predominant reactive species in conventional AOPs.^[^ ^22^ ^]^

The catalytic performance of SACs is strongly influenced by the electronic structure of the metal active center, which can be regulated by the coordination environment (e.g., coordination atoms and coordination numbers).^[^ ^23^ ^]^ Doping heteroatoms into the coordination sphere of the metal active site is an effective strategy for regulating the electronic structure of the metal center, thereby activating the metal active site.^[^ ^24^, ^25^ ^]^ For instance, doping oxygen (O) into Co‐based SACs can alter the electronic structure of Co active centers, facilitating the generation of ^1^O_2_.^[^ ^26^ ^]^ Modulating the electronic structure of single metal atoms through heteroatom doping is expected to promote the formation of high‐valent metal‐oxygen species, as demonstrated by B‐doping in the carbon substrates of Cu single‐atom catalysts.^[^ ^6^ ^]^ Furthermore, controlling heteroatoms to remotely modulate the SACs substrate can serve as an effective mechanism for tuning the electronic structure of metal centers.^[^ ^27^, ^28^ ^]^ Phosphorus (P), which shares the same number of valence electrons as nitrogen, often exhibits similar chemical properties. However, its larger atomic radius and higher electron‐donating ability make it a valuable choice for tuning the electronic density of metal centers.^[^ ^29^, ^30^ ^]^ Therefore, replacing N atoms of substrate with P is expected to enhance the formation of high‐valent metal‐oxygen species by remotely modulating the electronic structure of the metal centers.

This study presents an electronic regulation strategy to enhance high‐valent Co–oxo species (Co(IV)═O) generation efficiency in Co‐based single‐atom catalysts (SACs), improving the catalytic performance of Co‐based catalysts/PMS for the removal of recalcitrant pollutants. Phosphorus (P) was introduced to replace nitrogen (N) atoms coordinating with the Co active site at meta‐positions on the graphitic carbon nitride (g‐C_3_N_4_) support, resulting in a P‐doped Co SACs (Co─N_6_/C─P). This modification induced a positive shift in the Co atom's d‐band center, promoting electron transfer from the Co atom to neighboring atoms and lowering the energy barrier for Co(IV)═O formation. As a result, the steady‐state concentration of Co(IV)═O in the (Co─N_6_/C─P)/PMS system was 2.7 times higher than in the (Co─N_6_/C)/PMS system (without P doping), leading to a threefold increase in the kinetic constant for contaminant degradation and a 3.5‐fold increase in turnover frequency (TOF). The (Co─N_6_/C─P)/PMS system also significantly reduced cytotoxicity and genotoxicity in secondary effluents, owing to the high oxidation of Co(IV)═O to various contaminants. This work offers mechanistic insights for designing catalysts that efficiently generate high‐valent metal‐oxygen species, offering a promising approach for the removal of recalcitrant pollutants and toxicity reduction.

## 2 Results and Discussion

### 2.1 Remote Modulation in Single‐Atom Catalyst Design

**Figure 1:**
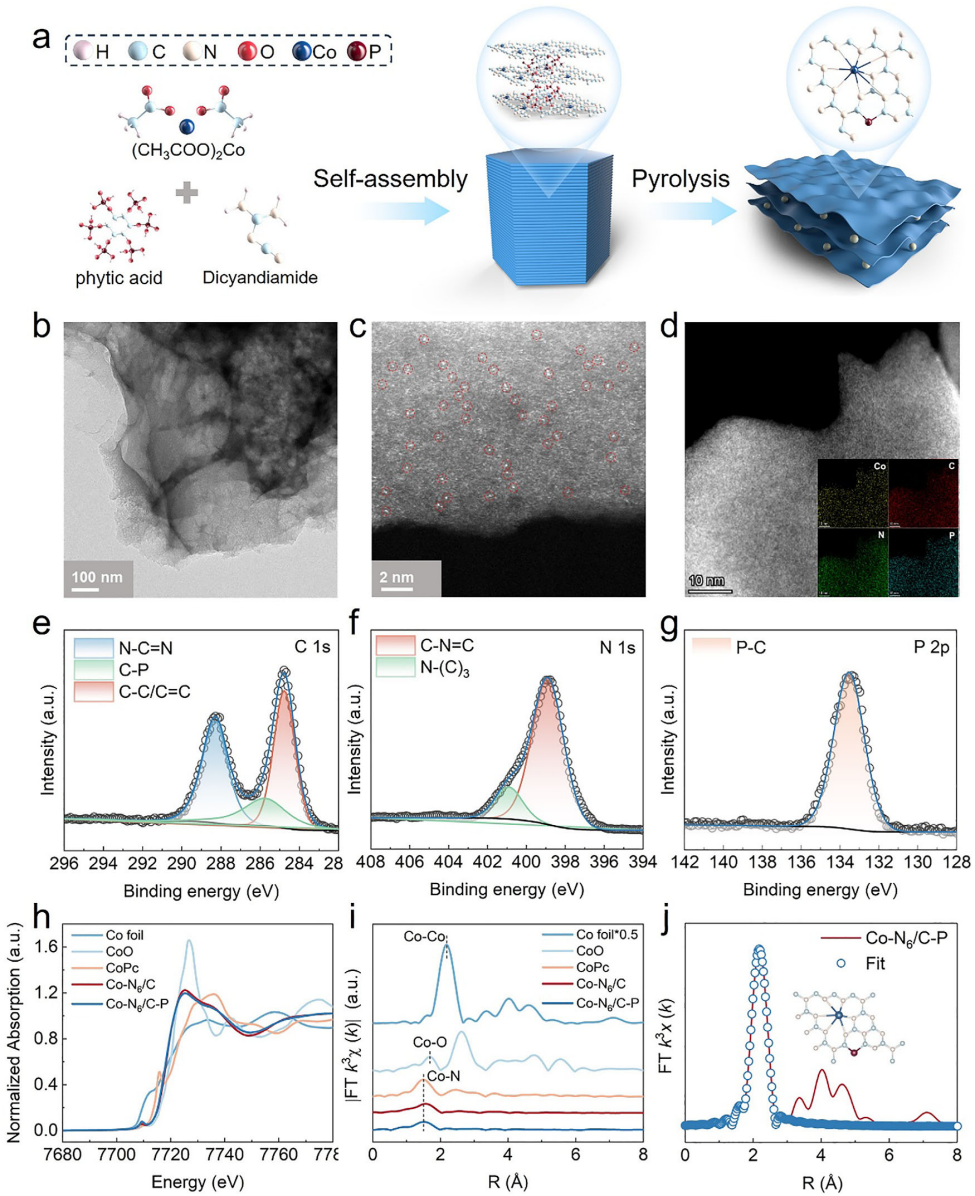
Synthesis method, characterizations, and atomic structure of Co─N_6_/C─P. a) Schematic diagram of Co─N_6_/C─P synthesis process. b) High‐magnification TEM images. c,d) High‐resolution HAADF‐STEM image and the corresponding EDS mapping image. e–g) C 1s, N 1s, and P 2p XPS spectra. h) Normalized Co K‐edge XANES spectra of Co─N_6_/C, Co─N_6_/C─P, and reference samples. i) Co K‐edge FT‐EXAFS spectra for Co─N_6_/C, Co─N_6_/C─P, and reference samples. j) Co K‐edge EXAFS fitting analysis for Co─N_6_/C─P.

A facile one‐step thermal polymerization method was employed to synthesize Co─N_6_/C─P (**Figure** **1**a). The Co‐containing precursor, phytic acid as a P source, and dicyandiamide as a carbon nitride precursor self‐assembled through hydrogen bonding, followed by pyrolyzed under a N_2_ atmosphere to anchor P‐doped Co SASs on the g‐C_3_N_4_ matrix. The transmission electron microscopy (TEM) images of the catalysts were used to determine the microscopic morphologies, elemental distribution, and atomic‐scale dispersion of the Co metal centers of the catalysts (Figure 1b). The Co─N_6_/C─P and Co─N_6_/C catalysts appeared as typical thin‐layer structures with no apparent metal nanoparticles or clusters. Isolated bright spots were clearly observed in the High‐angle annular dark field scanning transmission electron microscopy (HAADF‐STEM) image of Co─N_6_/C─P (Figure 1c), confirming the atomic dispersion of Co in Co─N_6_/C─P. The energy dispersive spectrometer (EDS) mapping of Co–N_6_/C─P showed a uniform distribution of various elements, including Co, C, N, and P, on the catalyst surface (Figure 1d). The ICP‐OES results revealed a Co content of 3.54% for Co─N_6_/C─P and 3.92% for Co─N_6_/C (Table S1, Supporting Information). Similar X‐ray diffraction (XRD) patterns were obtained for Co─N_6_/C and Co─N_6_/C─P that containing a clear diffraction peak at 27.2°. This peak is characteristic of the (002) plane of g‐C_3_N_4_ and indicates a periodic interlayer stacking structure (Figure S1, Supporting Information).^[^ ^31^ ^]^ The characteristic diffraction peaks of crystalline Co or Co oxide species were not observed, indicating the absence of metal clusters and oxides in Co─N_6_/C─P, which further confirmed the dispersion of Co at the atomic level. The specific surface area of Co─N_6_/C─P (20.37 m^2^/g) was greater than that of Co─N_6_/C (12.65 m^2^ g^−1^), which indicated that introducing P atoms into the framework made the architecture more porous (Figure S2, Supporting Information).^[^ ^32^ ^]^

The X‐ray photoelectron spectroscopy (XPS) spectra were analyzed to determine the elemental composition and states of Co─N_6_/C─P. The peak in the high‐resolution spectrum of C 1s could be divided into a C─C/C═C peak at 283.7 eV, a C─P peak at 284.7 eV, and a peak for a sp^2^ hybridized C from a triazine ring at 287.2 eV (Figure 1e).^[^ ^33^, ^34^ ^]^ The N 1s peak was divided into two peaks at 398.9 eV and 400.9 eV, corresponding to pyridinic N and graphitic N, respectively (Figure 1f). Only one peak of P 2p appeared at 133.5 eV, which was attributed to covalent bonding of P and C (Figure 1g).^[^ ^35^ ^]^ The proportion of Co^2+^ increased from 58.54% in Co─N_6_/C to 82.81% in Co─N_6_/C─P, suggesting that P‐doping promotes the conversion of Co^3+^ to Co^2+^ and forms Co(II)─N species through coordination with N atoms (Figure S3, Supporting Information).^[^ ^36^ ^]^

The chemical state and coordination structure of Co at the atomic level were investigated using XAFS. The normalized X‐ray absorption near‐edge structure (XANES) spectrum of the Co K‐edge revealed that the Co atoms in Co─N_6_/C─P were positively charged with valence states close to +2, as evidenced by the position of the Co‐rising edge between the Co foil and CoO being closer to CoO than Co (Figure 1h). This finding was consistent with the observations for Co─N_6_/C, indicating that the electronic structure of the Co atom was more affected by P doping than the valence of the Co atom. Fourier‐transformed *k^3^*‐weighted extended XAFS (FT‐EXAFS) was employed to further explore the bonding between the Co atoms and neighboring elements in Co─N_6_/C─P (Figure 1i). A strong peak appeared at 1.50 Å, which overlapped considerably with the peak corresponding to the Co─N bond in phthalocyanine Co, indicating the first shell of Co─N.^[^ ^37^ ^]^ No characteristic peak of Co─Co at ≈2.18 Å was detected, which proved the absence of crystalline Co species in Co─N_6_/C─P.^[^ ^38^ ^]^ The FT‐EXAFS spectra of the Co K‐edge could be effectively fitted to the scattering path of Co─N, suggesting that Co was only coordinated in Co─N_6_/C and Co─N_6_/C─P in the form of Co─N bonds (Figure 1j; Figure S4, Supporting Information). Consistent with the FT results, the maximum intensity in the wavelet transform diagram of Co─N_6_/C─P appeared at the Co─N site (Figure S5, Supporting Information). Moreover, the coordination number of the first shell Co─N was 5.7, and the average bond length was ≈2.02 Å (Table S2, Supporting Information). Therefore, it was deduced that P was doped into the g‐C_3_N_4_ matrix without changing the coordination structure of the Co active center. However, P doping may have influenced the electronic configuration of the Co active center through indirect coordination (inset of Figure 1j).

### 2.2 Catalytic Performance of (Co─N_6_/C─P)/PMS

**Figure 2:**
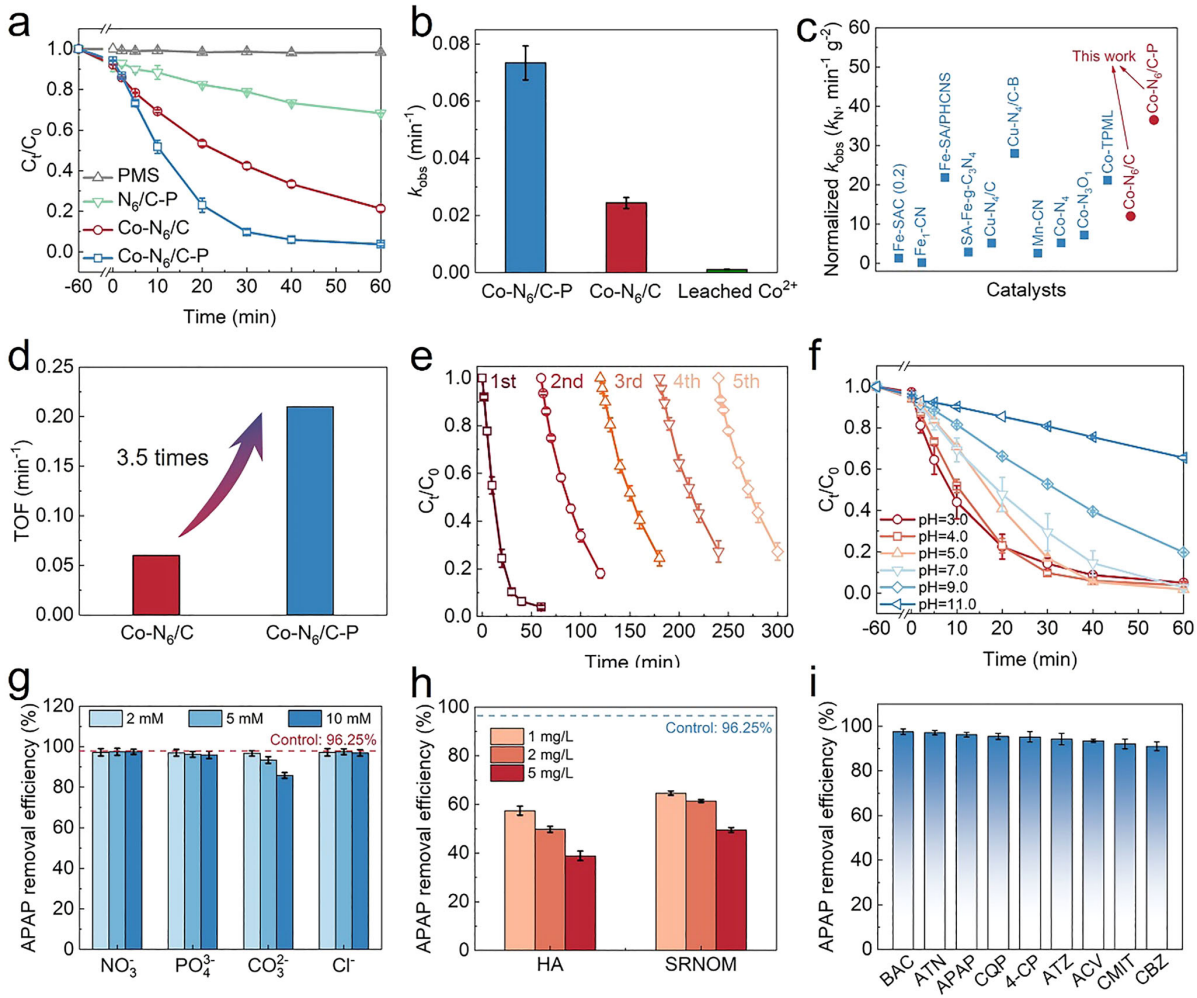
Catalytic performance of (Co─N_6_/C─P)/PMS system. a) APAP degradation by different catalyst‐activated PMS systems. b) *k*_obs_ of APAP degradation by (Co─N_6_/C)/PMS, (Co─N_6_/C─P)/PMS, and leached Co^2+^ activated PMS systems. c) Kinetic comparison of contaminants degradation by catalyst‐activated PMS systems in this study and previous literature. d) TOF of Co in (Co‐N_6_/C)/PMS and (Co‐N_6_/C‐P)/PMS. e) APAP degradation in five consecutive cycles. f) Influence of pH on APAP degradation. g) Influence of co‐existing anions on APAP degradation. h) Influence of co‐existing organic matters on APAP degradation. i) Degradation of multiple contaminants. Reaction condition: [catalyst] = 0.1 g L^−1^, [PMS] = 0.1 mM, [contaminants] = 2 mg L^−1^, initial pH 4.37 (if not adjusted).

The catalytic performance of various catalysts for activating PMS to degrade organic contaminants was investigated using acetaminophen (APAP) as a model. All the tested catalysts exhibited negligible adsorption of APAP during the first 60 min of reaction (**Figure** **2**a). Using PMS alone and (N_6_/C─P)/PMS had little effect on APAP degradation. The highest catalytic activity was observed for Co─N_6_/C─P, which achieved an APAP degradation efficiency of 96.25% within 60 min. The apparent reaction rate constant (*k*_obs_) of (Co─N_6_/C─P)/PMS (0.073 min^−1^) was approximately three times that of (Co─N_6_/C)/PMS (0.024 min^−1^) (Figure 2b; Figure S6, Supporting Information). The normalized *k_obs_* of (Co─N_6_/C─P)/PMS were considerably higher than most reported values, indicating highly efficient catalytic activity for Co─N_6_/C─P (Figure 2c; Table S3, Supporting Information). The TOF of Co in (Co─N_6_/C─P)/PMS (0.21 min^−1^) was 3.5 times that of (Co─N_6_/C)/PMS (0.06 min^−1^) (Figure 2d). These results suggested that P doping enhanced the activation of PMS to degrade APAP by the Co‐based SACs. Throughout the experimental process, the leaching concentration of Co^2+^ in the (Co─N_6_/C─P)/PMS system was detected at 3.02 µg L^−1^. This value is considerably lower than the reclaimed water limit set by the US Environmental Protection Agency (50 µg L^−1^), indicating the stability and safety of Co–N_6_/C─P used for water purification. The leached Co^2+^ had an extremely low *k_obs_* (0.001 min^−1^) for the degradation of APAP, accounting for only 1.39% of the total *k_obs_* (0.073 min^−1^) (Figure 2b; Figure S7, Supporting Information). It could thus be speculated that the Co atom was stably dispersed on the P‐doped g‐C_3_N_4_ matrix and functioned as an active site. Therefore, Co─N_6_/C─P could be considered a promising candidate for PMS activation. The degradation intermediates of APAP were identified using high‐resolution mass spectrometry (Table S4, Supporting Information). Based on these results, the possible degradation pathways involve hydroxylation and cleavage of the C─N bond (Figure S8, Supporting Information).

A five‐cycle experiment was conducted to test the reusability of Co─N_6_/C─P. The degradation efficiency of APAP remained above 72.75% after five cycles (Figure 2e), suggesting good activation performance and reusability for Co─N_6_/C─P. The degradation efficiency of (Co─N_6_/C─P)/PMS remained high over a wide pH range of 3.0–7.0 (Figure 2f). The degradation of APAP by (Co─N_6_/C─P)/PMS was clearly enhanced in acidic and neutral environments, but was somewhat inhibited at pHs exceeding 9.0. The negative effect of high pHs on APAP degradation could be attributed to the surface repulsion between the anionic forms of APAP, PMS, and Co─N_6_/C─P.^[^ ^21^, ^39^ ^]^ More OH^−^ was introduced as the pH increased, leading to quenching of the reactive species.^[^ ^40^ ^]^

The effect of various inorganic anions and organic matters on APAP degradation by (Co─N_6_/C─P)/PMS was investigated. The presence of NO_3_^−^ and Cl^−^ had a negligible effect on APAP degradation by (Co─N_6_/C─P)/PMS (Figure 2g and Figure S9, Supporting Information). However, PO_4_^3−^ and CO_3_^2−^ species slightly interfered with the reaction due to the quenching effect of additive anions on radicals.^[^ ^5^ ^]^ The representative dissolved organic matter used (natural organic matter from Suwannee River, SRNOM) and humic acid (HA) strongly inhibited APAP degradation by (Co─N_6_/C─P)/PMS. This inhibition effect became more pronounced with increasing concentration of organic matter (Figure 2h; Figure S10, Supporting Information). The inhibition of organic matters on APAP degradation by (Co─N_6_/C─P)/PMS could be ascribed to the comprehensive effect of quenching oxidatively reactive species, occupation of metal active sites on the catalyst surface, and competitive consumption of PMS.^[^ ^41^, ^42^ ^]^ The (Co─N_6_/C─P)/PMS system was used to degrade various organic contaminants, including benzalkonium chloride (BAC), atenolol (ATN), APAP, chloroquine diphosphate (CQP), 4‐chlorophenol (4‐CP), atrazine (ATZ), aciclovir (ACV), methylchloroisothiazolinone (CMIT), carbamazepine (CBZ). The (Co─N_6_/C─P)/PMS catalyst achieved a degradation efficiency of above 91% for the aforementioned organic contaminants within 60 min (Figure 2i; Figure S11, Supporting Information). These findings support the broad applicability of (Co─N_6_/C─P)/PMS to water purification.

### 2.3 Identification and Quantification of Reactive Species for (Co─N_6_/C─P)/PMS

**Figure 3:**
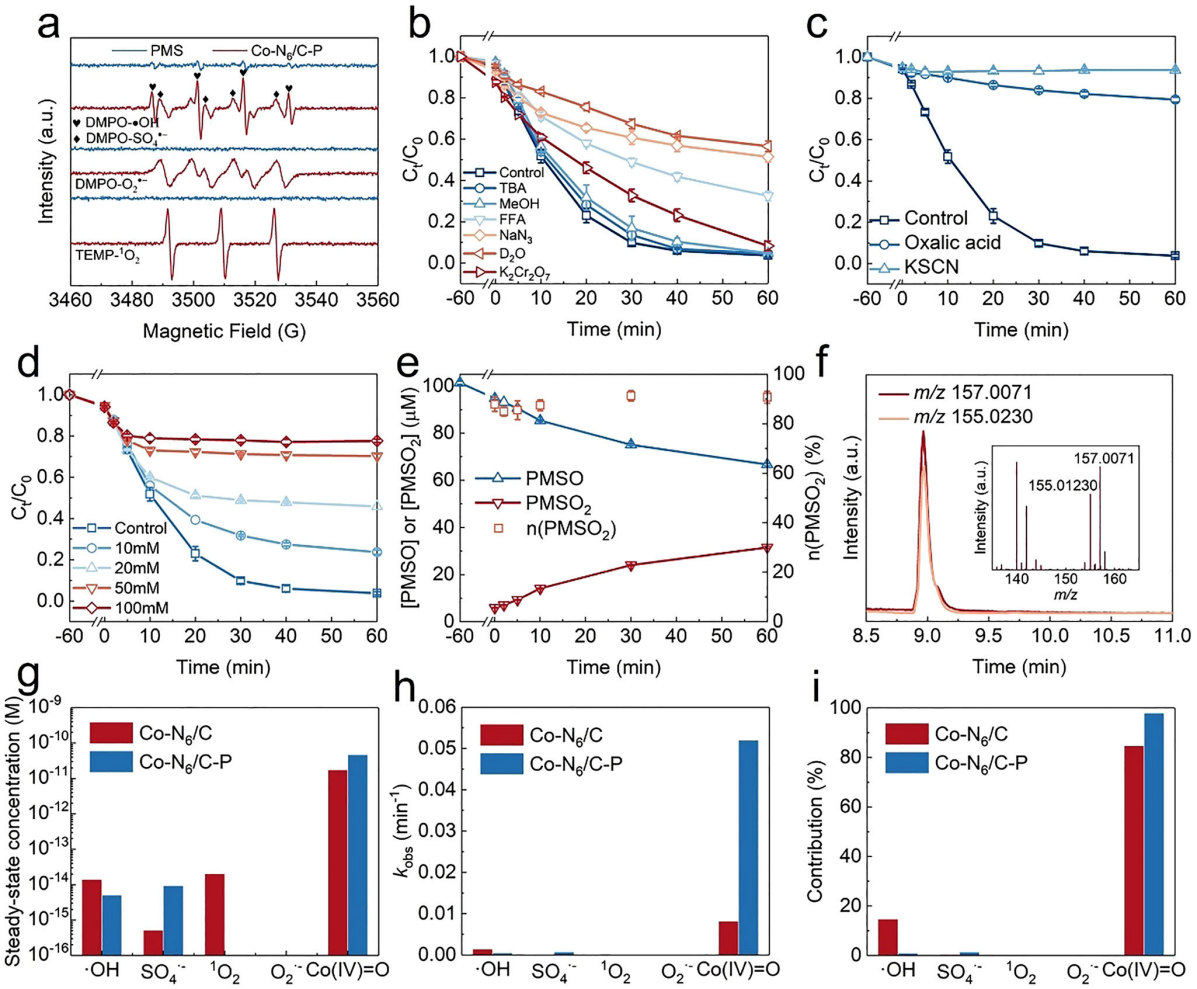
Roles of the reactive species in the (Co─N_6_/C─P)/PMS system. a) EPR spectra of the different systems captured by DMPO and TEMP. b) Quenching experiments of the (Co─N_6_/C─P)/PMS system. c) Influence of KSCN (25 mM) and oxalate (25 mm) on the APAP degradation. d) Influence of the DMSO concentration on the APAP degradation. e) Conversion of PMSO to PMSO_2_. f) Extracted ion chromatogram of PMS^16^O^16^O and PMS^16^O^18^O. g–i) Steady‐state concentrations, observed reaction rate constant, and oxidation contributions of different reactive species for APAP degradation. Reaction condition: [catalyst] = 0.1 g L^−1^, [PMS] = 0.1 mm, [APAP] = 2 mg L^−1^, initial pH 4.37.

The electron paramagnetic resonance (EPR) spectra and radical quenching experiments were employed to determine the dominant reactive species generated in the (Co─N_6_/C─P)/PMS system.^[^ ^43^ ^]^ 5,5‐Dimethyl‐1‐pyrroline *N*‐oxide (DMPO) was used as a spin trapping agent for ^•^OH, SO_4_^•−^ and O_2_^•−^ radicals, while 2,2,6,6‐tetramethylpiperidine (TEMP) was used as a spin trapping agent for ^1^O_2_. The EPR spectra contained characteristic peaks of DMPO‐^•^OH, DMPO‐SO_4_^•−^, TEMP‐^1^O_2_, and DMPO‐O_2_^•−^, indicating the generation of various reactive species, such as ^•^OH, SO_4_^•−^, ^1^O_2_, and O_2_^•−^, in the (Co─N_6_/C─P)/PMS system (**Figure** **3**a). The radicals generated by the (Co─N_6_/C─P)/PMS system were identified through quenching tests, using the second‐order reaction rate constants of the quenchers provided in Table S5 (Supporting Information). The addition of *tert*‐Butanol (TBA, a quencher for ⋅OH) and methanol (MeOH, a quencher for ⋅OH and SO_4_^•−^) slightly decreased the degradation efficiency of APAP from 96.25% to 95.47% and 95.21%, respectively, suggesting that ^•^OH and SO_4_^•−^ played unimportant roles in APAP degradation (Figure 3b). Both furfuryl alcohol (FFA, a quencher for ⋅OH and ^1^O_2_) and NaN_3_ (a quencher for ⋅OH, ^1^O_2_, and O_2_^•−^) inhibited APAP degradation. However, exchanging the solvent (H_2_O to D_2_O) to extend the ^1^O_2_ lifetime did not promote APAP degradation, revealing that ^1^O_2_ was not the main reactive species. The similar inhibitory effects of FFA and NaN_3_ showed that O_2_^•−^ played a negligible role in the degradation process. The relatively low second‐order reaction rate constants for the reaction between O_2_^•−^ and APAP (2.7 × 10^3^ M^−1^ s^−1^) also provide evidence of the negligible role of O_2_^•−^ in APAP degradation. Therefore, it could be concluded that SO_4_^•−^, ⋅OH, ^1^O_2_, and O_2_^•−^ were not the main reactive species for APAP degradation. Using K_2_Cr_2_O_7_ as an electrical scavenger had almost no effect on APAP degradation by the (Co─N_6_/C─P)/PMS system, indicating that the reaction did not involve oxidative degradation by electron transfer.^[^ ^6^ ^]^

Notably, adding oxalic acid and potassium thiocyanate (KSCN) to the (Co─N_6_/C─P)/PMS system considerably reduced the APAP degradation efficiency from 96.25% to 20.66% and 6.29%, respectively, supporting the conclusion that the active center of Co─N_6_/C─P is the Co atom and not the C atom or the doped heteroatom (Figure 3c). Using dimethyl sulfoxide (DMSO) (*k*_DMSO_, _Co(IV) = O_ = 2.4 × 10^6^ M^−1^ s^−1^) as a quencher, the APAP degradation efficiency decreased from 96.25% to 22.39% as the DMSO concentration was increased from 0 to 100 mm (Figure 3d). This suggests that Co(IV)═O is the main reactive species, which also demonstrates a strong oxidation effect on other pollutants (Figure S12, Supporting Information). Furthermore, methyl phenyl sulfoxide (PMSO) was employed as a chemical probe to investigate the generation of Co(IV)═O, because PMSO can be selectively converted to methyl phenyl sulfone (PMSO_2_) through an O‐atom transfer mechanism (*k*_PMSO, Co(IV) = O_ = 2 × 10^6^ M^−1^ s^−1^).^[^ ^44^, ^45^ ^]^ After 60 min of APAP degradation by the (Co─N_6_/C─P)/PMS system, the PMSO concentration decreased by 34.75 µm and the PMSO_2_ concentration increased by 31.52 µm, corresponding to a conversion efficiency of over 84.93% (Figure 3e). This remarkable finding suggests that a large quantity of Co(IV)═O was generated in the (Co─N_6_/C─P)/PMS system and played a primary role in APAP degradation. Moreover, ^18^O‐isotope‐labeled H_2_O (H_2_^18^O) was used as a solvent to detect the generation of Co(IV)═O, as Co(IV)═O could spontaneously exchange O atoms with a solvent (the reaction principle is shown in Figure S13, Supporting Information).^[^ ^46^, ^47^ ^]^ The extracted ion chromatogram of the (Co─N_6_/C─P)/PMS system with H_2_^18^O as the solvent contained peaks of both PMS^16^O^16^O at *m*/*z* 155.0230 and PMS^16^O^18^O at *m*/*z* 157.0071 (Figure 3f). In summary, all the findings presented above show that Co(IV)═O was the main reactive species generated in the (Co─N_6_/C─P)/PMS system.

The steady‐state concentrations of the reactive species (^•^OH, SO_4_^•−^, ^1^O_2_, O_2_^•−^, and Co(IV)═O) were measured using benzoic acid (BA), nitrobenzene (NB), FFA, *p*‐chlorobenzoic acid (*p*CBA), and PMSO as specific probes, respectively. For quantification purposes, the probe concentrations used were an order of magnitude lower than that of the target contaminant APAP to ensure that the probes had almost no effect on APAP degradation (Figure S14, Supporting Information). The steady‐state concentration and contribution of each reactive species were determined through competitive kinetics using the rate constants for the second‐order reaction between the probes and reactive species (Figure S15 and Table S6, Supporting Information). The steady‐state concentration of Co(IV)═O (>10^−11^ m) was considerably higher than those of ^•^OH, SO_4_^•−^, ^1^O_2_, and O_2_^•−^ (<10^−14^ m) in both the (Co─N_6_/C)/PMS and (Co─N_6_/C─P)/PMS systems (Figure 3g). Thus, the main reactive species was identified as Co(IV)═O. The steady‐state concentration of Co(IV)═O was 4.76 × 10^−11^ m in the (Co─N_6_/C─P)/PMS system, which was 2.7 times higher than that in the (Co─N_6_/C)/PMS system (1.75 × 10^−11^ m). The corresponding *k_obs_* for the oxidative contribution from Co(IV)═O increased from 8.22 × 10^−3^ min^−1^ (84.74% of the total contribution) in the (Co─N_6_/C)/PMS system to 5.20 × 10^−2^ min^−1^ (97.95% of the total contribution) in the (Co─N_6_/C─P)/PMS system (Figure 3h,i). Therefore, P‐doping enabled efficient generation and increased the oxidative contribution of Co(IV)═O, improving the catalytic performance of the Co‐based SACs.

### 2.4 Mechanism of P‐doping Regulation

**Figure 4:**
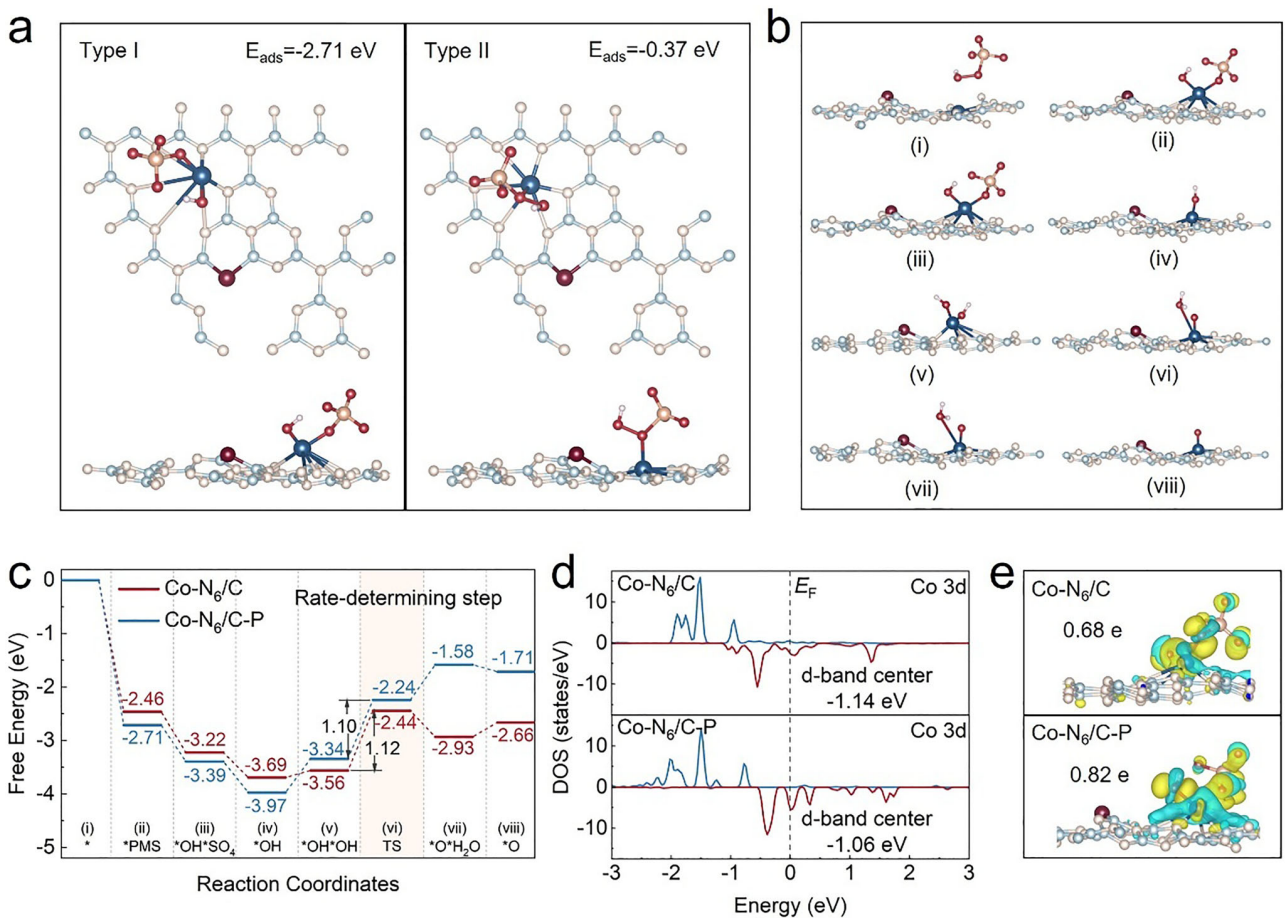
Theoretical study of the catalyst mechanism. a) Two conceivable adsorption configurations between Co─N_6_/C─P and PMS. b) The structures of reaction intermediates for the generation of Co(IV)═O. c) Energy profiles of Co(IV)═O formation for (Co─N_6_/C)/PMS and (Co─N_6_/C─P)/PMS systems; ^*^O represents the reactive species of Co(IV)═O. d) PDOS of Co in Co─N_6_/C and Co─N_6_/C─P. e) Charge‐density differences of Co─N_6_/C and Co─N_6_/C─P, where blue and yellow represented electron depletion and electron accumulation, respectively.

Density functional theory (DFT) calculations were conducted to analyze the regulation of P‐doping engineering on the electronic structures of the Co‐based SACs. Two configurations for PMS adsorption on the catalyst surface were considered: coordination of the Co atom to the O atom of the O─O bond adjacent to the H atom (Type I) or sulfur (S) atom (Type II) (**Figure** **4**a). The adsorption energy was more negative for the Type I configuration (−2.71 eV) than the Type II configuration (−0.37 eV), revealing that PMS was more likely to be adsorbed on the Co─N_6_/C─P surface in a Type I configuration. The strong adsorption capacity of PMS caused the O─O bond in PMS to elongate to its breaking point, leading to co‐adsorption of OH and SO_4_ groups on the surface of the Co center site, which was highly conducive to further activation of PMS.^[^ ^13^ ^]^ Notably, the O─O bond length in the Type II configuration remained at 1.48 Å, indicating that the interaction of Co─N_6_/C─P with PMS via Type II adsorption was insufficient to directly cleave the O─O bond.^[^ ^13^, ^36^ ^]^ These calculation results suggested that PMS most likely adsorbed onto Co─N_6_/C─P in the Type I configuration.

The free energies of Co(IV)═O generated on Co─N_6_/C and Co─N_6_/C─P were compared to elucidate the mechanism by which P‐doping facilitated the selective generation of Co(IV)═O. A mechanism was proposed for the generation of Co(IV)═O on the catalyst surface consisting of PMS adsorption followed by a series of reactions: ^*^ i) → ^*^PMS ii) → ^*^OH^*^SO_4_ iii) → ^*^OH iv) → ^*^OH^*^OH v) → transition state (TS, vi) → ^*^O^*^H_2_O vii) → ^*^O viii), where ^*^ represents the adsorption state and ^*^O represents Co(IV)═O (Figure 4b). The Gibbs free energy of Co(IV)═O generation was −2.66 eV on Co─N_6_/C, and −1.71 eV on Co─N_6_/C─P, suggesting this process was both thermodynamically feasible and kinetically favorable (Figure 4c). The adsorption of PMS on Co–N_6_/C─P (−2.71 eV) is significantly stronger than on Co─N_6_/C (−2.46 eV), indicating that P‐doping enhances PMS adsorption and facilitates its activation. Remarkably, the highest reaction energy barrier was determined for the formation of the ^*^O^*^H_2_O vi) intermediate, which was therefore identified as the rate‐determining step for Co(IV)═O generation. This energy barrier decreased from 1.12 eV for (Co─N_6_/C)/PMS to 1.10 eV for (Co─N_6_/C─P)/PMS. Therefore, P‐doping facilitated the formation of Co(IV)═O by enhancing PMS adsorption on the catalyst and lowering the energy barrier for the formation of the ^*^O^*^H_2_O transition state.

The partial density of states (PDOS) was calculated to explore the effect of P‐doping on the electron distribution of the Co active center. The d‐band center was calculated as −1.06 eV for Co─N_6_/C─P, and −1.14 eV for Co─N_6_/C (Figure 4d), suggesting that P‐doping brought the d‐band center of the Co active center closer to the Fermi level (E_F_). Thus, more antibonding states were occupied, which enhanced the adsorption of PMS and facilitated the formation of key reaction intermediates (^*^PMS).^[^ ^48^, ^49^ ^]^ At the same time, the narrowing of the gap between the d‐band center and E_F_ increased the charge rate at the reaction interface, reducing the activation energy (E_a_) and free energy barrier (ΔG). Bader charge analysis proved that P‐doping enhanced electron transfer from Co atoms to adjacent atoms (0.82 e in Co─N_6_/C─P versus 0.68 e in Co─N_6_/C) (Figure 4e), thereby reducing the Co site charge density and increasing the positive electrostatic potential, which in turn enhanced the electrostatic adsorption of PMS on Co─N_6_/C─P.^[^ ^50^ ^]^ Therefore, it could be concluded that P‐doping optimized the electron distribution of the Co 3d orbitals and reduced the electron density of the Co active centers. This modulation enhanced PMS adsorption on Co‐based SACs and lowered the energy barrier for the formation of key reaction intermediates of Co(IV) = O, facilitating the rapid and selective generation of Co(IV)═O.

### 2.5 Applications of the Catalyst for Real Wastewater Treatment

**Figure 5:**
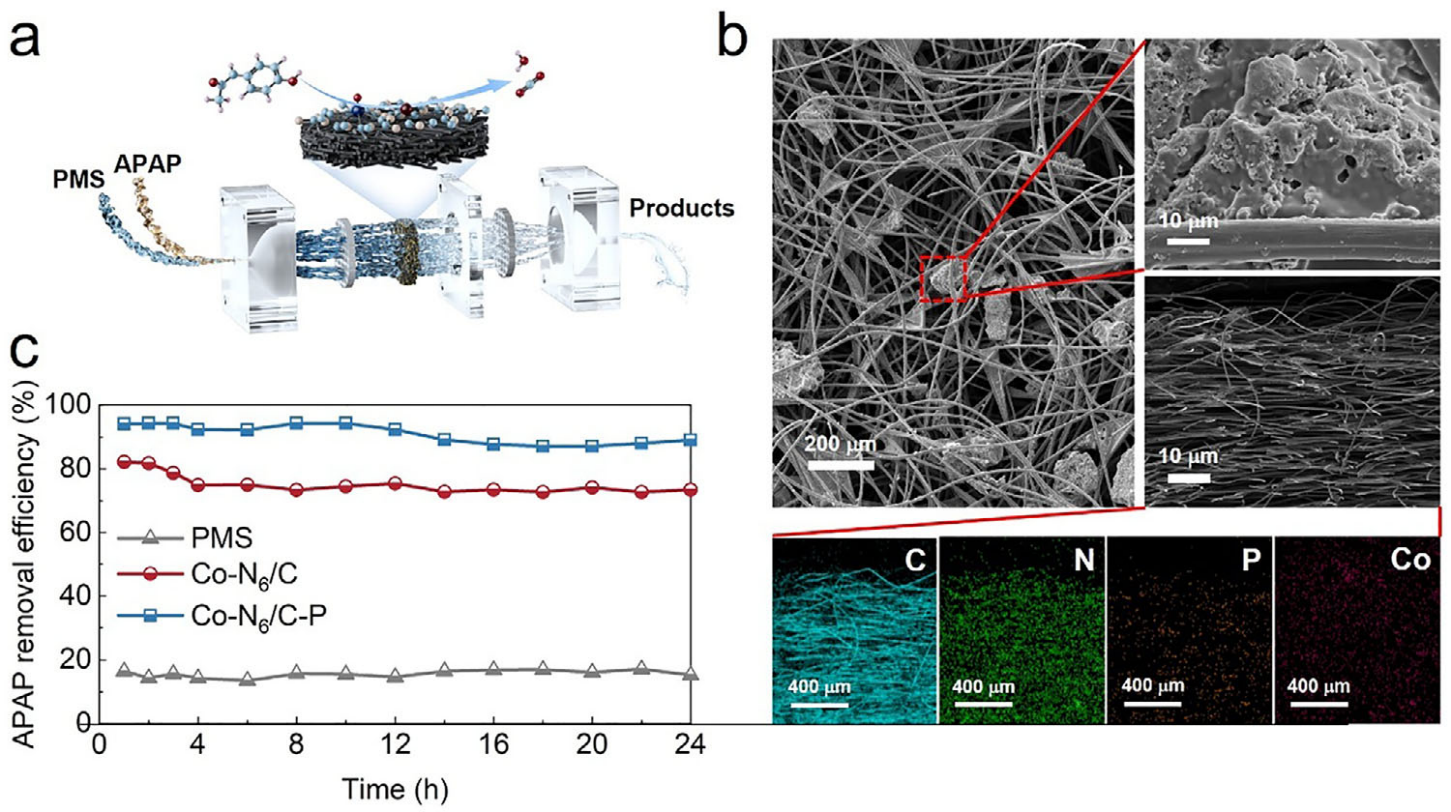
Water purification device integration and continuous operation. a) The schematic illustration of the continuous flow system. b) SEM images of carbon felt loaded with Co─N_6_/C─P. c) Continuous operation test of APAP degradation in the continuous flow system. Reaction condition: Flow rate = 2 mL min^−1^, Catalyst loading = 50 mg (if needed), [PMS] = 0.5 mM, [APAP] = 1 mg L^−1^, HRT = 1.23 min.

A continuous‐flow reactor was used to evaluate the practical applicability of Co─N_6_/C─P (**Figure** **5**a). Considering the high surface area and porosity of Co─N_6_/C─P determined from the SEM images (Figure S16, Supporting Information), carbon felt was used as a support to facilitate the rapid permeation of water.^[^ ^51^, ^52^ ^]^ The Co─N_6_/C─P catalyst was loaded onto the carbon felt as the core reaction unit. SEM images of the loaded carbon felt revealed that Co─N_6_/C─P was uniformly dispersed on the surface (Figure 5b), providing sufficient contact for pollutants in the treated water. A cross‐sectional SEM image and EDS mapping of the loaded carbon felt revealed that the deposited Co─N_6_/C─P layer was 400‐µm thick. Treated wastewater was collected from the secondary effluent of a municipal wastewater treatment plant, and APAP was added as a typical pharmaceutical pollutant to investigate APAP degradation in real wastewater. A continuous‐flow experiment was conducted for 24 h using a water flux of 245 L m^−2^ h^−1^. During 24 h of continuous operation, the carbon‐felt filters loaded with Co─N_6_/C─P maintained an APAP removal efficiency of over 87%, which was markedly higher than that of Co─N_6_/C (72%, Figure 5c). In contrast, <17% APAP removal occurred using PMS/carbon felt. Moreover, the leached Co ions concentrations (18.6–32.1 µg L^−1^, Figure S17, Supporting Information) in the carbon felt ─Co─N_6_/C─P/PMS system were below the U.S. Environmental Protection Agency's reclaimed water limit (50 µg L^−1^), indicating a strong interaction between the catalyst and the support. Therefore, the (Co─N_6_/C─P)/carbon felt exhibited high activity for PMS activation as well as excellent stability and safety for water purification applications.

**Figure 6:**
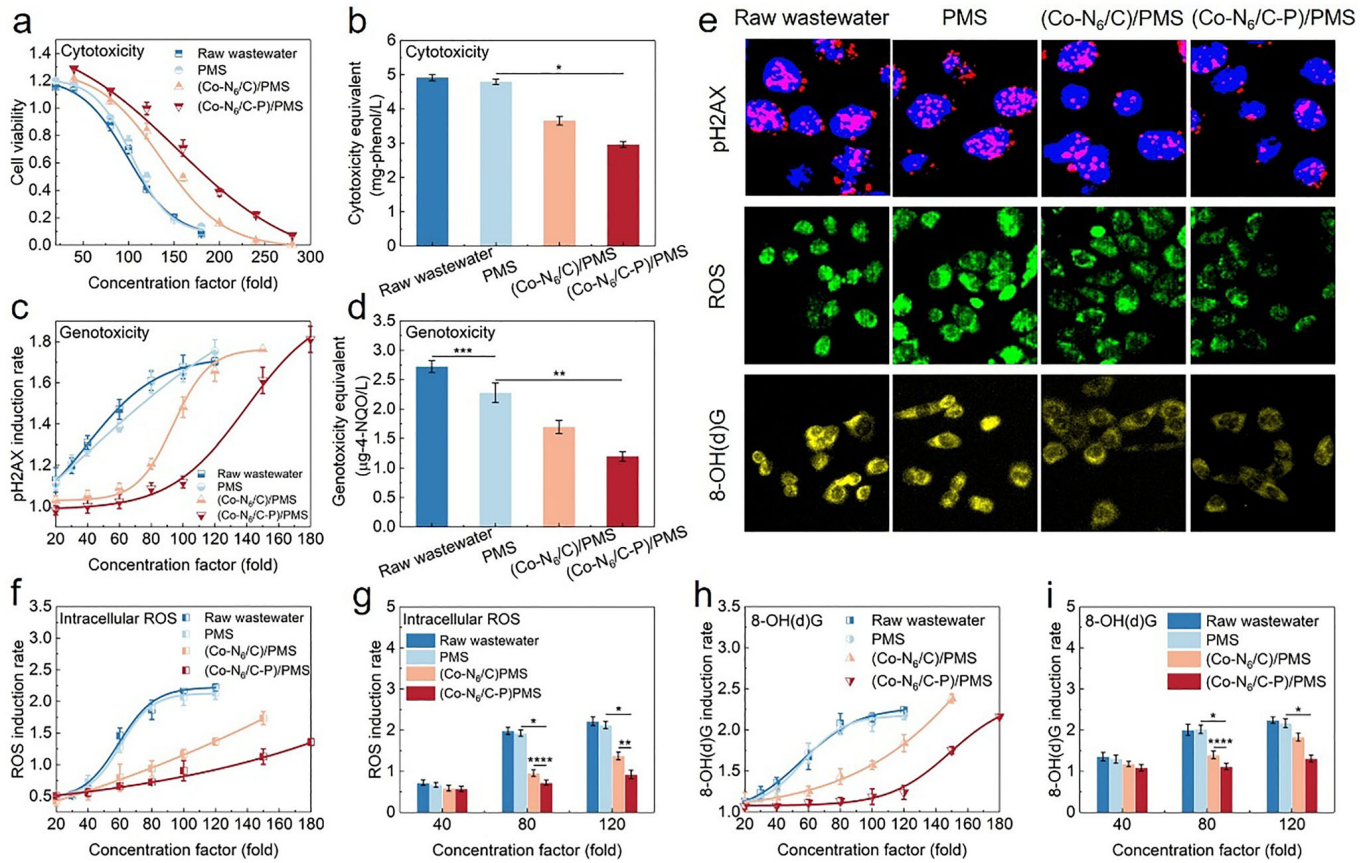
Toxicity changes of the practical wastewater after oxidation processes. a) concentration–effect curves of cytotoxicity. b) Cytotoxicity equivalents. c) Fluorescence images of intracellular pH2AX foci (red), ROS (green), and 8‐OH(d)G (yellow) in CHO cells. d) Concentration–effect curves of genotoxicity. e) Genotoxicity equivalents. f,g) Intracellular ROS induction rate. h,i) 8‐OH(d)G induction rate. “^*^” means *p* < 0.05, “^**^” means *p* < 0.01, and “^***^” means *p* < 0.001, accessed by one‐way analysis of variance. Reaction condition: [catalyst] = 0.1 g L^−1^, [PMS] = 0.1 mm, [APAP] = 2 mg L^−1^.

Real wastewater has various components and may form toxic byproducts during degradation that pose potential environmental risks. Chinese hamster ovary (CHO) cells were used as models to assess the toxicity of treated wastewater to mammals, which is practically important. The organic contaminants in the collected secondary effluent could be categorized into five groups, including aromatic protein tyrosine‐like (I), aromatic protein tryptophan‐like (II), fulvic acid‐like (III), soluble microbial by‐product‐like (IV), and humic acid‐like (V) (Figure S18 and Table S7, Supporting Information). In the raw wastewater, the organic contaminants in Category II had the highest fluorescence intensity, followed by those in Categories III, IV, V, and I, showing that these substances were cytotoxic and genotoxic.^[^ ^53^ ^]^ The (Co─N_6_/C)/PMS and (Co─N_6_/C─P)/PMS systems considerably reduced the cytotoxicity of the wastewater (**Figure** **6**a). By contrast, using PMS alone had little impact on the wastewater cytotoxicity. The wastewater cytotoxicity was quantified by calculating cytotoxicity equivalents from concentration–effect curves. The (Co─N_6_/C─P)/PMS system lowered the wastewater cytotoxicity from 4.92 to 2.96 mg‐phenol L^−1^, outperforming the (Co─N_6_/C)/PMS system, which reduced the wastewater cytotoxicity to 3.66 mg‐phenol L^−1^ (Figure 6b). Therefore, the (Co─N_6_/C─P)/PMS system has a good capacity for cytotoxicity removal, which may result from the strong oxidative capability of Fe(IV)═O.

The genotoxicity of the treated wastewater was assessed by characterizing and quantifying the DNA double‐strand break (DSB) using phosphorylated H2AX histone (pH2AX). The most severe form of DNA damage is considered to be a DNA DSB, which induces pH2AX to recruit proteins and facilitate the repair of damaged DNA.^[^ ^54^ ^]^ Therefore, pH2AX is directly correlated with DNA DSBs and is a marker of genetic toxicity. Similar to the cytotoxicity results, the genotoxicity was considerably reduced by the (Co─N_6_/C─P)/PMS system from 2.72 to 0.08 µg‐4‐NQO L^−1^ (Figure 6c,d). The (Co─N_6_/C)/PMS system was comparatively less effective and reduced the wastewater genotoxicity from 2.72 to 0.11 µg‐4‐NQO L^−1^. Adding PMS alone to the wastewater only slightly lowered the genotoxicity from 2.72 to 2.28 µg‐4‐NQO L^−1^. The quantity of pH2AX in the cell nucleus (claret foci) decreased after treating the wastewater with the (Co─N_6_/C─P)/PMS system, confirming the reduction in the genotoxicity (Figure 6e).

Cytotoxicity and genotoxicity might be caused by increased intracellular oxidative stress, which is related to the excessive induction of intracellular reactive oxygen species (ROS).^[^ ^55^, ^56^ ^]^ In addition, ROS‐induced DNA/RNA oxidative damage manifests as base modifications at the C‐8 position of deoxyguanosine and guanosine, corresponding to the production of 8‐hydroxy‐deoxyguanosine and 8‐hydroxyguanosine (collectively referred to as 8‐OH(d)G). Treating the wastewater with the (Co─N_6_/C)/PMS and (Co─N_6_/C─P)/PMS systems considerably decreased the levels of both intracellular ROS and 8‐OH(d)G (Figure 6f–i), where (Co─N_6_/C─P)/PMS was more efficient than (Co─N_6_/C)/PMS. This difference in efficiency became more pronounced as the concentration factor increased. Fluorescent analysis was also employed to evaluate the levels of intracellular ROS and 8‐OH(d)G, in which a stronger fluorescent signal represented a higher level (Figure 6e). The fluorescence images revealed that the cells in the water samples treated with (Co─N_6_/C)/PMS and (Co─N_6_/C─P)/PMS systems produced weak fluorescent signals, indicating low levels of intracellular ROS and 8‐OH(d)G. Overall, the in vitro studies clearly demonstrated that the toxicity of wastewater can be reduced through treatment by the (Co─N_6_/C─P)/PMS system. Reducing the levels of harmful contaminants considerably increases water safety.

## 3 Conclusion

In this study, meta‐site modulation of a Co single‐atom catalyst was engineered through P‐doping (Co─N_6_/C─P) to enhance PMS activation over Co‐based SAC catalysts. This strategy involved using P atoms to substitute N atoms coordinated with a Co center at meta‐positions, which remotely modulated the electronic structure of the Co active center. As a result, the charge density of the Co site decreased, and the d‐band center of the Co atom shifted positively, lowering the energy barrier for Co(IV)═O generation. Remote modulation via P doping ensured the efficient generation of Co(IV)═O and effective conversion of Co active sites within the Co─N_6_/C─P/PMS system, thereby enhancing the sustainable removal of persistent contaminants. The (Co─N_6_/C─P)/PMS system exhibits excellent potential for wastewater purification and offers the following advantages: i) applicability over a wide pH range of 3–7, ii) good reusability, iii) robustness to complex water matrices, and iv) near‐complete elimination of various micropollutants.

The secondary effluent was effectively treated using a continuous flow reactor based on the (Co─N_6_/C─P)/PMS system. Over 87% contamination removal was achieved during 24 h of continuous and stable operation. The (Co─N_6_/C─P)/PMS system significantly reduced the toxicity of the secondary effluent, including cytotoxicity, genotoxicity, and levels of intracellular ROS and 8‐OH(d)G. These results demonstrate that the (Co─N_6_/C─P)/PMS system offers dual benefits in water purification by efficiently removing persistent contaminants and reducing toxicity. The novel technology developed in this study enhances the selective generation of Co(IV)═O through remote modulation of metal active sites, presenting a promising strategy for ensuring water quality and safety.

## Conflict of Interest

The authors declare no conflict of interest.

## Supporting information



Supporting Information

## Data Availability

The data that support the findings of this study are available in the supplementary material of this article.

## References

[1] E. Svensson Grape , A. J. Chacón‐García , S. Rojas , Y. Pérez , A. Jaworski , M. Nero , M. Åhlén , E. Martínez‐Ahumada , A. E. Galetsa Feindt , M. Pepillo , M. Narongin‐Fujikawa , I. A. Ibarra , O. Cheung , C. Baresel , T. Willhammar , P. Horcajada , A. K. Inge , Nat. Water. 2023, 1, 433.

[2] S. S. Lau , K. Bokenkamp , A. Tecza , E. D. Wagner , M. J. Plewa , W. A. Mitch , Nat. Sustain. 2022, 6, 39.

[3] C. H. Gu , Y. Pan , T. T. Wei , A. Y. Zhang , Y. Si , C. Liu , Z. H. Sun , J. J. Chen , H. Q. Yu , Nat. Water. 2024, 2, 649.

[4] S. Wei , Y. Sun , Y. Z. Qiu , A. Li , C. Y. Chiang , H. Xiao , J. Qian , Y. Li , Nat. Commun. 2023, 14, 7549. 37985662 10.1038/s41467-023-43040-5 PMC10662205

[5] Z. Wang , E. Almatrafi , H. Wang , H. Qin , W. Wang , L. Du , S. Chen , G. Zeng , P. Xu , Angew. Chem., Int. Ed. 2022, 61, 202202338. 10.1002/anie.202202338 35514041

[6] X. Zhou , M. K. Ke , G. X. Huang , C. Chen , W. Chen , K. Liang , Y. Qu , J. Yang , Y. Wang , F. Li , H. Q. Yu , Y. Wu , Proc. Natl. Acad. Sci. USA 2022, 119, 2119492119. 10.1073/pnas.2119492119 PMC8872710 35165185

[7] Q. Zhou , C. Song , P. Wang , Z. Zhao , Y. Li , S. Zhan , Proc. Natl. Acad. Sci. USA 2023, 120, 2300085120. 10.1073/pnas.2300085120 PMC10068799 36952382

[8] J. Shi , Y. Wei , D. Zhou , L. Zhang , X. Yang , Z. Miao , H. Qi , S. Zhang , A. Li , X. Liu , W. Yan , Z. Jiang , A. Wang , T. Zhang , ACS Catal. 2022, 12, 7760.

[9] Z. Q. Zhang , P. J. Duan , J. X. Zheng , Y. Q. Xie , C. W. Bai , Y. J. Sun , X. J. Chen , F. Chen , H. Q. Yu , Nat. Commun. 2025, 16, 115. 39747208 10.1038/s41467-024-55622-y PMC11697253

[10] Z. Guo , Y. Xie , J. Xiao , Z. J. Zhao , Y. Wang , Z. Xu , Y. Zhang , L. Yin , H. Cao , J. Gong , J. Am. Chem. Soc. 2019, 141, 12005. 31276405 10.1021/jacs.9b04569

[11] Q. Y. Wu , Z. W. Yang , Z. W. Wang , W. L. Wang , Proc. Natl. Acad. Sci. USA 2023, 120, 2219923120.

[12] X. Liang , D. Wang , Z. Zhao , T. Li , Y. Gao , C. Hu , Adv. Funct. Mater. 2022, 32, 2203001.

[13] C. Chu , J. Yang , X. Zhou , D. Huang , H. Qi , S. Weon , J. Li , M. Elimelech , A. Wang , J. H. Kim , Environ. Sci. Technol. 2021, 55, 1242. 33213138 10.1021/acs.est.0c06086

[14] Q. Wang , C. Liu , D. Zhou , X. Chen , M. Zhang , K. Lin , Chem. Eng. J. 2022, 439, 135002.

[15] X. Liang , D. Wang , Z. Zhao , T. Li , Z. Chen , Y. Gao , C. Hu , Appl. Catal., B 2022, 303, 120877.

[16] H. Dong , Y. Li , S. Wang , W. Liu , G. Zhou , Y. Xie , X. Guan , Environ. Sci. Technol. Lett. 2020, 7, 219.

[17] Z. Yang , J. Qian , A. Yu , B. Pan , Proc. Natl. Acad. Sci 2019, 116, 6659. 30872470 10.1073/pnas.1819382116 PMC6452667

[18] H. Li , C. Shan , B. Pan , Environ. Sci. Technol. 2018, 52, 2197. 29373017 10.1021/acs.est.7b05563

[19] H. Chen , Y. Xu , K. Zhu , H. Zhang , Appl. Catal., B 2021, 284, 119732.

[20] P. Yang , S. Li , L. Xiaofu , A. Xiaojing , D. Liu , W. Huang , Sep. Purif. Technol. 2022, 285, 120288.

[21] Z. Wang , W. Wang , J. Wang , Y. Yuan , Q. Wu , H. Hu , Appl. Catal., B 2022, 305, 121049.

[22] J. Lee , U. von Gunten , J. H. Kim , Environ. Sci. Technol. 2020, 54, 3064. 32062964 10.1021/acs.est.9b07082

[23] Z. Wang , E. Almatrafi , H. Wang , H. Qin , W. Wang , L. Du , S. Chen , G. Zeng , P. Xu , Angew. Chem., Int. Ed. 2022, 202202338. 10.1002/anie.202202338 35514041

[24] M. Fan , J. Cui , J. Wu , R. Vajtai , D. Sun , P. M. Ajayan , Small 2020, 16, 1906782. 10.1002/smll.201906782 32363806

[25] S. Zha , D. Wang , C. Liu , W. Wang , N. Mitsuzaki , Z. Chen , Sustain. Energy Fuels 2022, 6, 3895.

[26] X. Zhou , M. K. Ke , G. X. Huang , C. Chen , W. Chen , K. Liang , Y. Qu , J. Yang , Y. Wang , F. Li , H. Q. Yu , Y. Wu , Proc. Natl. Acad. Sci 2022, 119, 2119492119. 10.1073/pnas.2119492119 PMC8872710 35165185

[27] W. Ni , Y. Gao , Y. Zhang , H. A. Younus , X. Guo , C. Ma , Y. Zhang , J. Duan , J. Zhang , S. Zhang , ACS Appl. Mater. Interfaces 2019, 11, 45825. 31702129 10.1021/acsami.9b18510

[28] H. Shen , E. Gracia‐Espino , J. Ma , K. Zang , J. Luo , L. Wang , S. Gao , X. Mamat , G. Hu , T. J. A. C. I. E. Wagberg , Angew. Chem., Int. Ed. 2017, 56, 13800. 10.1002/anie.201706602 28857381

[29] C. Saka , Appl. Catal., B 2021, 292, 120165.

[30] D. S. Yang , D. Bhattacharjya , S. Inamdar , J. Park , J. S. Yu , J. Am. Chem. Soc. 2012, 134, 16127. 22966761 10.1021/ja306376s

[31] Y. Kang , Y. Yang , L. C. Yin , X. Kang , G. Liu , H. M. Cheng , Adv. Mater. 2015, 27, 4572. 26149596 10.1002/adma.201501939

[32] J. Di , J. Xia , M. Ji , B. Wang , S. Yin , Q. Zhang , Z. Chen , H. Li , Appl. Catal., B 2016, 183, 254.

[33] Y. Gao , Y. Zhu , L. Lyu , Q. Zeng , X. Xing , C. Hu , Environ. Sci. Technol. 2018, 52, 14371. 30424598 10.1021/acs.est.8b05246

[34] H. Wang , Y. Cai , Z. Zhang , Y. Liu , R. Zhang , R. Huo , R. Wu , H. Cheng , J. Yin , J. Yin , Z. Su , H. Zhu , J. Energy Storage 2025, 110, 115329.

[35] X. Zhu , T. Zhang , D. Jiang , H. Duan , Z. Sun , M. Zhang , H. Jin , R. Guan , Y. Liu , M. Chen , H. Ji , P. Du , W. Yan , S. Wei , Y. Lu , S. Yang , Nat. Commun. 2018, 9, 4177. 30301894 10.1038/s41467-018-06437-1 PMC6177470

[36] B. Liu , W. Guo , W. Jia , H. Wang , Q. Si , Q. Zhao , H. Luo , J. Jiang , N. Ren , Environ. Sci. Technol. 2021, 55, 12640. 34464118 10.1021/acs.est.1c04091

[37] Z. Wang , Y. Wang , W. Wang , D. Wu , Q. Wu , H. Hu , Appl. Catal., B 2023, 324, 122248.

[38] H. Fei , J. Dong , Y. Feng , C. S. Allen , C. Wan , B. Volosskiy , M. Li , Z. Zhao , Y. Wang , H. Sun , P. An , W. Chen , Z. Guo , C. Lee , D. Chen , I. Shakir , M. Liu , T. Hu , Y. Li , A. I. Kirkland , X. Duan , Y. Huang , Nat. Catal. 2018, 1, 63.

[39] Y. Qi , J. Li , Y. Zhang , Q. Cao , Y. Si , Z. Wu , M. Akram , X. Xu , Appl. Catal., B 2021, 286, 119910.

[40] X. Wu , K. Rigby , D. Huang , T. Hedtke , X. Wang , M. W. Chung , S. Weon , E. Stavitski , J. H. Kim , Environ. Sci. Technol. 2022, 56, 1341. 34964609 10.1021/acs.est.1c06371

[41] C. Guan , J. Jiang , C. Luo , S. Pang , Y. Yang , Z. Wang , J. Ma , J. Yu , X. Zhao , Chem. Eng. J. 2018, 337, 40.

[42] Y. Meng , Z. Li , J. Tan , J. Li , J. Wu , T. Zhang , X. Wang , Chem. Eng. J. 2022, 429, 130860.

[43] L. Wang , X. Lan , W. Peng , Z. Wang , J. Hazard. Mater. 2021, 408, 124436. 33191023 10.1016/j.jhazmat.2020.124436

[44] C. Cheng , W. Ren , F. Miao , X. Chen , X. Chen , H. Zhang , Angew. Chem., Int. Ed. 2023, 62, 202218510. 10.1002/anie.202218510 36625681

[45] Z. Wang , J. Jiang , S. Pang , Y. Zhou , C. Guan , Y. Gao , J. Li , Y. Yang , W. Qiu , C. Jiang , Environ. Sci. Technol. 2018, 52, 11276. 30207707 10.1021/acs.est.8b02266

[46] Y. Zong , Y. Shao , Y. Zeng , B. Shao , L. Xu , Z. Zhao , W. Liu , D. Wu , Environ. Sci. Technol. 2021, 55, 7634. 33706511 10.1021/acs.est.1c00375

[47] B. Huang , Z. Wu , X. Wang , X. Song , H. Zhou , H. Zhang , P. Zhou , W. Liu , Z. Xiong , B. Lai , Environ. Sci. Technol. 2023, 57, 15667. 37801403 10.1021/acs.est.3c05509

[48] Y. Zhang , X. Chen , C. Liang , L. Yin , Y. Yang , Appl. Catal., B 2022, 315, 121536.

[49] K. Qian , H. Chen , W. Li , Z. Ao , Y. Wu , X. Guan , Environ. Sci. Technol. 2021, 55, 7034. 33620197 10.1021/acs.est.0c08805

[50] Y. Zou , J. Hu , B. Li , L. Lin , Y. Li , F. Liu , X. Li , Appl. Catal., B 2022, 312, 121408.

[51] J. Xu , X. Zheng , Z. Feng , Z. Lu , Z. Zhang , W. Huang , Y. Li , D. Vuckovic , Y. Li , S. Dai , G. Chen , K. Wang , H. Wang , J. K. Chen , W. Mitch , Y. Cui , Nat. Sustain. 2020, 4, 233. 34355066 10.1038/s41893-020-00635-w PMC8330436

[52] P. Duan , J. Pan , W. Du , Q. Yue , B. Gao , X. Xu , Appl. Catal., B 2021, 299, 120714.

[53] Y. Du , W. L. Wang , Z. W. Wang , C. J. Yuan , M. Q. Ye , Q. Y. Wu , Environ. Sci. Technol. 2023, 57, 3311. 36787277 10.1021/acs.est.2c06965

[54] R. Anand , A. Beach , K. Li , J. Haber , Nature 2017, 544, 377. 28405019 10.1038/nature22046 PMC5544500

[55] Y. Du , Y. Yang , W.‐L. Wang , Y.‐T. Zhou , Q. Y. Wu , Environ. Int. 2020, 135, 105369. 31841803 10.1016/j.envint.2019.105369

[56] Z. Wang , W. Wang , J. Wang , D. Wang , M. Liu , Q. Wu , H. Hu , Adv. Funct. Mater. 2022, 33, 2209560.

