# Supplementary material for: Remote Modulation of Single‐Atom Catalyst Boosts High‐Valent Cobalt–Oxo Species Generation for Water Purification and Detoxification
Source: Adv Sci (Weinh). 2025 Dec 12;13(11):e12498. doi: 10.1002/advs.202512498 (PMC12931198; doi:10.1002/advs.202512498)
Supplement: Supplementary file 1 — Supporting Information [file ADVS-13-e12498-s001.pdf]

Supporting Information

Remote Modulation of Single-Atom Catalyst Boosts High-Valent Cobalt–Oxo Species Generation for Water Purification and Detoxification

Wen-Min Wang[1, 2], Zheng-Wei Yang[1, 2], De-Xiu Wu[1, 2], Wen-Long Wang[1, 2], Qian-Yuan Wu[1, 2, *]

Affiliations:

[1]Shenzhen Key Laboratory of Ecological Remediation and Carbon Sequestration, Environmental Protection Key Laboratory of Microorganism Application and Risk Control, Institute of Environment and Ecology, Shenzhen International Graduate School, Tsinghua University, Shenzhen 518055, PR China

[2]Key Laboratory of Microorganism Application and Risk Control of Shenzhen, Guangdong Provincial Engineering Research Center for Urban Water Recycling and Environmental Safety, Institute of Environment and Ecology, Shenzhen International Graduate School, Tsinghua University, Shenzhen 518055, PR China

*Corresponding Author. Email: wu.qianyuan@sz.tsinghua.edu.cn

Table of Contents

Experimental Section

Supporting Figures (S1-S18)

Supporting Tables (S1-S7)

References

Experimental Section

Materials and Chemicals

For chemical analysis, Ultrapure water (18.2 MΩ·cm) was prepared by a water purifier system (Milli-Q Integral). Meroxymonosulfate (PMS, $KHSO_5 \cdot 0.5KHSO_4 \cdot 0.5K_2SO_4$) was obtained from Meryer Reagent Co., Ltd., Shanghai, China. Acetaminophen (APAP), dicyandiamide, oxalic acid and potassium dichromate ($K_2Cr_2O_7$) were purchased from Rhawn Reagent Co., Ltd, Shanghai, China. Cobalt acetate ($Co(CH_3COO)_2$), furfuryl alcohol (FFA), dimethyl sulfoxide (DMSO), methyl phenyl sulfoxide (PMSO), methyl phenyl sulfone ($PMSO_2$), 4-chlorophenol (4-CP), methylchloroisothiazolinone (CMIT), nitrobenzene (NB), *tert*-Butanol (TBA), *p*-benzoquinone (PBQ), sodium azide (NaN_3), D_2O, $H_2{}^{18}O$ and sodium hydroxide (NaOH were purchased from Macklin Co., Ltd., Shanghai, China. Potassium thiocyanate (KSCN), ascorbic acid, formic acid, chloroquine diphosphate (CQP), aciclovir (ACV), *p*-chlorobenzoic acid (*p*CBA) and benzoic acid (BA) were obtained from Aladdin Bio-Tech (Shanghai, China). Benzalkonium chloride (BAC), atenolol (ATN), atrazine (ATZ), and carbamazepine (CBZ) were obtained from J&K (Beijing, China). Methanol and acetonitrile were all of chromatographic grade (J.T. Baker, New Jersey, U.S.A.).

For toxicity assays, methanol, acetone, and dichloromethane were all of chromatographic grade (J.T. Baker, New Jersey, U.S.A.). Phenol, 4-nitroquinoline N-oxide (4-NQO), Hoechst 33342, and paraformaldehyde were obtained from Sigma Aldrich (St. Louis, MO, USA). Triton X-100 (reagent grade) was purchased from Solarbio (Beijing, China). Phosphate-buffered saline (Hyclone), a mixture of Dulbecco's Modified Eagle Medium and F-12 (DMEM/F-12 1:1 medium), and penicillin–streptomycin were bought from Thermo Fisher Scientific (Waltham, MA, USA). Bovine serum albumin (BSA) was purchased from Amresco (Solon, OH, USA). The kit for cytotoxicity assay (Cell Counting Kit-8, CCK-8) was obtained from Dojindo (Kumamoto, Japan). Triton X-100 and Albumin Bovine V were purchased from Solarbio (China). Paraformaldehyde and Hoechst 33342 were bought from Sigma-Aldrich (U.S.A). Primary antibody phospho-Histone H2AX (S139) and secondary

antibody Alexa Fluor 647 conjugate for genotoxicity assay were purchased from Cell Signaling Technology (Boston, MA, USA).

Synthesis of Catalysts

The P-doped Co single-atom catalyst (Co–N_6/C–P) was synthesized by a facile one-step thermal polymerization process using dicyandiamide as a precursor of the CN matrix, $Co(CH_3COO)_2$ as a metal source, and phytic acid as a P source. First, 5 g of dicyandiamide and 3.5 mL of phytic acid were dissolved in 40 mL of ultrapure water (UPW). Next, 500 mg of $Co(CH_3COO)_2$ were added to and completely mixed with the resulting solution. The resulting mixture was heated at 70°C to remove water. The resulting solid was placed in a tube furnace with a set program to provide heating from room temperature to 600°C at a rate of 5°C/min and kept under a N_2 atmosphere for 3 h. The obtained product was ground sufficiently, immersed in 1 M H_2SO_4 for 6 h, and subsequently washed repeatedly with UPW until the scrubbing solution reached a neutral pH. Finally, the product was vacuum dried at 80°C for 12 h, and the catalyst was collected. The same method was used to prepare the Co single-atom catalyst (Co–N_6/C) and the P-doped single atom catalyst (N_6/C–P) in the absence of phytic acid and $Co(CH_3COO)_2$, respectively.

Characterization of Catalysts

Transmission electron microscopy (TEM, Tecnai G2 F30, FEI) was conducted at a 200-kV acceleration voltage to characterize the morphologies of the catalysts. High-angle annular dark field scanning transmission electron microscopy (HAADF-STEM) images were obtained by using the STEM mode of an FEI Themis Z with double aberration correctors. An energy dispersive spectrometer (EDS, EDS super-X) was used to observe the elemental composition and distribution of the catalysts. X-ray diffraction (XRD, Rigaku Smartlab) was employed to identify the crystal phases of the catalysts. X-ray photoelectron spectroscopy (XPS, Thermo Scientific K-Alpha) was used to analyze the surface elemental compositions and chemical states of the catalysts.

Electron paramagnetic resonance (EPR) spectra were obtained using a Bruker A300 spectrometer at room temperature. A Brunauer–Emmett–Teller (BET) surface area analyzer (ASAP 2020 Plus) was employed to determine the BET surface area, pore volume, and pore distribution of the catalysts. An elemental analyzer (EA, Elementar vario EL cube) was used to determine the O content of the catalysts. Inductively coupled plasma optical emission spectrometry (ICP-OES, Agilent 5110) was performed to measure the content of Co loaded into the catalysts. X-ray absorption fine structure (XAFS) spectra of the Co K-edge were recorded at beamline 7 to 3 of the Stanford Synchrotron Radiation Laboratory, and the data were processed using Athena software.

Activity of Catalyst

Batch experiments were conducted in 100-mL conical flasks to study acetaminophen (APAP) degradation in a catalyst/PMS system. In a typical experiment, the catalyst was first dispersed in a solution of the target organic contaminants for 60 min under stirring at 25°C to reach adsorption equilibrium. Next, a prescribed quantity of PMS stock was added to the aforementioned mixture to produce a solution with 2 mg/L organic contaminants, 0.1 mM PMS, and 0.1 g/L catalyst. A 1-mL of the supernatant was withdrawn at prescribed intervals, filtered through a 0.22-μm nylon membrane, and subsequently mixed with ascorbic acid (20 μL) to rapidly quench the reaction. The initial pH of the contaminant solution was adjusted by adding H_2SO_4 and NaOH solutions without any buffers to prevent obtaining misleading results. All experiments were performed in duplicate or triplicate.

Analytical Methods

The concentrations of the organic contaminants were determined using a high-performance liquid chromatograph (HPLC, Shimadzu) combined with a photodiode-array detector. Separation was performed on a C_{18} column (250 mm × 4.2 mm, 5 μm) using an injection volume of 20 μL. The PMS concentration was determined by the spectrophotometric method[1]. Ultra-high-performance liquid chromatography

(UHPLC, UltiMate 3000) coupled with a Q Exactive mass spectrometer (Thermo Scientific, USA) was used to determine the intermediate products formed during APAP degradation. Experiments on ^{18}O-isotope labeling were conducted in a $H_2{}^{18}O$ solvent, and $^{16}O/^{18}O$ isotope-labeled $PMSO_2$ was identified using a Thermo Q-Exactive Focus Orbitrap mass spectrometer operating in positive (+ESI) mode. The three-dimensional excitation-emission matrix of water samples were recorded using a fluorescence spectrophotometer (F-7000, Hitachi, Japan).

Calculation of Normalized k_{obs}

The reaction rates of various catalysts with the contaminants were evaluated using a modified kinetics model, as shown in the following equation:

$$k_N = \frac{k_{obs}}{C_{cat.} \times C_{PMS}}$$

where, k_{obs} (min^{-1}) is the observed reaction rate constant; k_N (min^{-1} g^{-2}) is the normalized k_{obs}; and $C_{cat.}$ (g/L) and C_{PMS} (g/L) are the dose of catalyst and PMS, respectively.

Calculation of Turnover Frequency (TOF)

TOF was introduced as a normalized indicator to quantitatively evaluate the enhancement of (Co-N_6/C-P)/PMS performance. The TOF of each Co active site was calculated as follows:

$$\mathrm{TOF} = \frac{k_{obs,\ APAP}}{m_{cat.} \times \eta_{cat.}}$$

where, $k_{obs,APAP}$ (min^{-1}) represents the pseudo-first reaction rate constant of APAP degradation; mcat. (g/L) represents the catalyst dosage; $\eta_{cat.}$ (wt%) represent the load amount of Co atom of catalyst.

Calculation of the Steady-state Concentration and Contributions of Reactive Species

The calculation of the steady-state concentration and contributions of reactive

species were reported in our previous study.[2]

(i) the steady-state concentrations of $^{\bullet}OH$, $SO_4^{\bullet-}$, $^1O_2$ and $O_2^{\bullet-}$

$$-\frac{d[BA]}{dt} = k_{BA,\,^{\bullet}OH}[^{\bullet}OH][BA] + k_{BA,\,SO_4^{\bullet-}}[SO_4^{\bullet-}][BA] \tag{S1}$$

$$-\frac{d[NB]}{dt} = k_{NB,\,^{\bullet}OH}[^{\bullet}OH][NB] + k_{NB,SO_4^{\bullet-}}[SO_4^{\bullet-}][NB] \tag{S2}$$

$$-\frac{d[pCBA]}{dt} = k_{pCBA,\,^{\bullet}OH}[^{\bullet}OH][pCBA] + k_{pCBA,SO_4^{\bullet-}}[SO_4^{\bullet-}][pCBA] + k_{pCBA,\,^1O_2}[^1O_2][pCBA] + k_{pCBA,O_2^{\bullet-}}[O_2^{\bullet-}][pCBA] \tag{S3}$$

$$-\frac{d[FFA]}{dt} = k_{FFA,\,^{\bullet}OH}[^{\bullet}OH][FFA] + k_{FFA,SO_4^{\bullet-}}[SO_4^{\bullet-}][FFA] + k_{FFA,\,^1O_2}[^1O_2][FFA] + k_{FFA,O_2^{\bullet-}}[O_2^{\bullet-}][FFA] \tag{S4}$$

Integrating Eqs. S1-S4,

$$-\ln\frac{[BA]}{[BA]_0} = (k_{BA,\,^{\bullet}OH}[^{\bullet}OH]_{ss} + k_{BA,SO_4^{\bullet-}}[SO_4^{\bullet-}]_{ss})t = k_{obs,BA}t \tag{S5}$$

$$-\ln\frac{[NB]}{[NB]_0} = (k_{NB,\,^{\bullet}OH}[^{\bullet}OH]_{ss} + k_{NB,SO_4^{\bullet-}}[SO_4^{\bullet-}]_{ss})t = k_{obs,NB}t \tag{S6}$$

$$-\ln\frac{[pCBA]}{[pCBA]_0} = (k_{pCBA,\,^{\bullet}OH}[^{\bullet}OH]_{ss} + k_{pCBA,SO_4^{\bullet-}}[SO_4^{\bullet-}]_{ss} + k_{pCBA,\,^1O_2}[^1O_2]_{ss} + k_{pCBA,O_2^{\bullet-}}[O_2^{\bullet-}]_{ss})t = k_{obs,pCBA}t \tag{S7}$$

$$-\ln\frac{[FFA]}{[FFA]_0} = (k_{FFA,\,^{\bullet}OH}[^{\bullet}OH]_{ss} + k_{FFA,SO_4^{\bullet-}}[SO_4^{\bullet-}]_{ss} + k_{FFA,\,^1O_2}[^1O_2]_{ss} + k_{FFA,O_2^{\bullet-}}[O_2^{\bullet-}]_{ss})t = k_{obs,FFA}t \tag{S8}$$

The relationship of $-\ln\frac{[BA]}{[BA]_0}$, $-\ln\frac{[NB]}{[NB]_0}$, $-\ln\frac{[pCBA]}{[pCBA]_0}$ and $-\ln\frac{[FFA]}{[FFA]_0}$ versus time were plotted and linearly fitted, and the resulting slopes were the pseudo-first-order reaction kinetic constants (i.e., $k_{obs,BA}$, $k_{obs,NB}$, $k_{obs,pCBA}$ and $k_{obs,FFA}$) for the degradation of the various probe compounds. Then, the concentrations of $^{\bullet}OH$ ($[^{\bullet}OH]_{ss}$), $SO_4^{\bullet-}$ ($[SO_4^{\bullet-}]_{ss}$), $^1O_2$ ($[^1O_2]_{ss}$) and $O_2^{\bullet-}$ ($[O_2^{\bullet-}]_{ss}$) were calculated using Eqs. S5-S8.

(ii) the steady-state concentration of Co(IV)=O

$$-\frac{d[PMSO_2]}{dt} = k_{PMSO,Co(IV)=O}[Co(IV)=O][PMSO] \tag{S9}$$

$$\eta = \frac{\Delta[\mathrm{PMSO_2}]}{\Delta[\mathrm{PMSO}]} = \frac{[\mathrm{PMSO_2}]}{[\mathrm{PMSO}]_0 - [\mathrm{PMSO}]} \tag{S10}$$

Associating Eqs. S9 and S10,

$$\frac{\mathrm{d}[\mathrm{PMSO_2}]}{[\mathrm{PMSO}]_0 - \frac{1}{\eta}[\mathrm{PMSO_2}]} = -k_{\mathrm{PMSO,Co(IV)=O}}[\mathrm{Co(IV)=O}]\mathrm{dt} \tag{S11}$$

Integrating Eq. S11,

$$\eta \ln \frac{[\mathrm{PMSO}]_0}{[\mathrm{PMSO}]_0 - \frac{1}{\eta}[\mathrm{PMSO_2}]} = k_{\mathrm{PMSO,Co(IV)=O}}[\mathrm{Co(IV)=O}]_{ss}t = k_{\mathrm{obs,PMSO_2}}t \tag{S12}$$

$$[\mathrm{Co(IV)=O}]_{ss} = \frac{k_{\mathrm{obs,PMSO_2}}}{k_{\mathrm{PMSO,Co(IV)=O}}} \tag{S13}$$

(iii) the contributions of reactive species

$$-\frac{\mathrm{d}[\mathrm{APAP}]}{\mathrm{dt}} = k_{\mathrm{APAP,^{\bullet}OH}}[^{\bullet}\mathrm{OH}][\mathrm{APAP}] + k_{\mathrm{APAP,SO_4^{\bullet-}}}[\mathrm{SO_4^{\bullet-}}][\mathrm{APAP}] + k_{\mathrm{APAP,^1O_2}}[^1\mathrm{O_2}][\mathrm{APAP}] + k_{\mathrm{APAP,O_2^{\bullet-}}}[\mathrm{O_2^{\bullet-}}][\mathrm{APAP}] + k_{\mathrm{APAP,Co(IV)=O}}[\mathrm{Co(IV)=O}][\mathrm{APAP}] \tag{S14}$$

Integrating Eq. S14,

$$-\ln\frac{[\mathrm{APAP}]}{[\mathrm{APAP}]_0} = (k_{\mathrm{APAP,^{\bullet}OH}}[^{\bullet}\mathrm{OH}]_{ss} + k_{\mathrm{APAP,SO_4^{\bullet-}}}[\mathrm{SO_4^{\bullet-}}]_{ss} + k_{\mathrm{APAP,^1O_2}}[^1\mathrm{O_2}]_{ss} + k_{\mathrm{APAP,O_2^{\bullet-}}}[\mathrm{O_2^{\bullet-}}]_{ss} + k_{\mathrm{APAP,Co(IV)=O}}[\mathrm{Co(IV)=O}]_{ss})t = k_{\mathrm{obs,APAP}}t \tag{S15}$$

The relationship of $-\ln\frac{[\mathrm{APAP}]}{[\mathrm{APAP}]_0}$ versus time was established and linearly fitted to derive apparent reaction rate constants for APAP degradation. The apparent reaction rate and oxidative contributions to APAP degradation of various reactive species are calculated as Eqs. S16-S21,

$$k_{\mathrm{obs,^{\bullet}OH}} = k_{\mathrm{APAP,^{\bullet}OH}}[^{\bullet}\mathrm{OH}]_{ss} \tag{S16}$$

$$k_{\mathrm{obs,SO_4^{\bullet-}}} = k_{\mathrm{APAP,SO_4^{\bullet-}}}[\mathrm{SO_4^{\bullet-}}]_{ss} \tag{S17}$$

$$k_{\mathrm{obs,^1O_2}} = k_{\mathrm{APAP,^1O_2}}[^1\mathrm{O_2}]_{ss} \tag{S18}$$

$$k_{\mathrm{obs,O_2^{\bullet-}}} = k_{\mathrm{APAP,O_2^{\bullet-}}}[\mathrm{O_2^{\bullet-}}]_{ss} \tag{S19}$$

$$k_{\mathrm{obs,Co(IV)=O}} = k_{\mathrm{obs,APAP}} - k_{\mathrm{obs,^{\bullet}OH}} - k_{\mathrm{obs,SO_4^{\bullet-}}} - k_{\mathrm{obs,^1O_2}} - k_{\mathrm{obs,O_2^{\bullet-}}} \tag{S20}$$

$$\text{Contribution (\%)} = \frac{k_{\text{obs,ROS}}}{k_{\text{obs,APAP}}} \times 100\% \tag{S21}$$

(iv) The second-order reaction rate between APAP and Co(IV)=O

$$k_{\text{APAP, Co(IV)=O}} = \frac{k_{\text{obs, Co(IV)=O}}}{[\text{Co(IV)=O}]_{\text{ss}}} \tag{S22}$$

Computational Methods

All the spin-polarized DFT calculations were performed using the Vienna Ab initio Simulation Program (VASP).[3] The generalized gradient approximation in the Perdew–Burke–Ernzerhof form and a cutoff energy of 500 eV for planewave basis set were adopted.[4] A 5 × 5 × 1 Monkhorst-Pack grid was used for sampling the Brillouin zones at structure calculation.[5] The ion-electron interactions were described by the projector augmented wave method.[6] The convergence criteria of structure optimization were chosen as a maximum force on each atom of less than 0.02 eV/Å with an energy change of less than 1 × 10^{-5} eV. To calculate the kinetic energy barriers of the chemical reactions, the climbing image nudged elastic band method was used to search for the transition states.[7] The Gibbs free energy (ΔG) for each elemental step was defined as follows[8]:

$$\Delta G = \Delta E_{\text{ads}} + \Delta E_{\text{zpe}} - T\Delta S + \Delta G_{\text{pH}}$$

where ΔE_{ads} and ΔE_{zpe} are the adsorption energy for the density functional theory calculations and the zero-point energy correction, respectively; and T, ΔS, and ΔG_{pH} are the temperature, entropy change, and free energy correction of the pH, respectively.

Solid Phase Extraction

The real wastewater samples used in this study were secondary effluents from a wastewater treatment plant in Shenzhen, China. First, 2 mg/L APAP was added to 1 L of a secondary effluent, followed by treatment with different catalyst/PMS systems. The organic matter in the treated water samples was enriched by solid-phase extraction

(SPE) for use in a toxicity assay. The SPE method was reported in our previous study.[9] Water samples were firstly acidified to pH=2 with H_2SO_4 (2 M), then passed through the extraction cartridges (Oasis hydrophilic lipophilic balance®, 6 mL, Waters, Milford, MA, U.S.A.) at a flow rate of 5 mL/min. The cartridges were activated by 10 mL methanol and 10 mL ultrapure water beforehand. Cartridges were then completely dried under nitrogen gas flow, then eluted with 5 mL methanol, 2 mL acetone and 2 mL dichloromethane in sequence. The eluents were completely dried under a stream of nitrogen and stored at -20°C for use. The SPE extracts were redissolved in 1 mL of DMEM/F-12 medium containing 0.5% (v/v) DMSO immediately before toxicity assay.

Cytotoxicity Assay

A Chinese hamster ovary cell line (CHO-K1) was obtained from the American Type Culture Collection. Streptomycin (0.1 mg/mL), penicillin G (100 unit/mL), and fetal bovine serum (10%) were added to a DMEM/F12 (1:1) culture medium. Cell culture dishes were placed in an incubator with saturated humidity and 5% CO_2 gas at 37 °C. Cells with two to five passages were used for the toxicity assay. Cell passaging was conducted every 48 h.

Cytotoxicity Assay and Cytotoxicity Equivalent

Cells were seeded in a sterile 96-well plate (Corning 3599, U.S.A.) and incubated for 24 h. The dried organic extracts were first dissolved in the culture medium containing 0.5% (v/v) dimethyl sulfoxide (DMSO), then further diluted to different concentration factors with the culture medium containing 0.5% DMSO. Cell cultures were exposed to organic extracts at different concentration factors for 48 h. The culture medium containing 0.5% DMSO was set as the negative control, while phenol dissolved in the culture medium containing 0.5% DMSO was used as the reference compound. After 48 h exposure, the culture medium was discarded and then washed

with 100 μL phosphate buffered solution (PBS). Then 100 μL of the reagent in Cell Counting Kit-8 (CCK-8) dissolved in DMEM/F12 was added into each well of the 96-well plates. Cells were then cultured in the incubator (37 °C) for another 2 h, then the absorbance of each well at 450 nm was measured using the microplate reader SpectraMax i3 (Molecular Devices, USA). Each test was performed in 4-8 replicates.

The cell viability (CV) for a sample or reference compound was calculated as follows:

$$CV=\frac{A_S-A_B}{A_N-A_B}$$

where CV is the cell viability for a sample or phenol against the negative control. The A_S is the absorbance of the sample or phenol at 450 nm. A_B is the absorbance of the blank control (only added the Cell Counting Kit-8 reagent dissolved in DMEM/F12) at 450 nm. The An is the absorbance of the negative control at 450 nm.

The CV values at different concentration factors of each sample were used to create a concentration–effect curve. Similarly, concentration–effect curve of phenol was obtained with the CV values at different concentrations. The concentration at which the CV value was 50% from the regression analysis was defined as the lethal concentration of 50% (LC_{50}). The cytotoxicity equivalent of a sample was calculated as follows:

$$\text{Cytotoxicity equivalent}=\frac{LC_{\text{Phenol},50}}{LC_{\text{Sample},50}}$$

where the cytotoxicity equivalent unit is mg–phenol/L. $LC_{\text{Phenol},50}$ is the LC_{50} of the reference compound phenol. $LC_{\text{sample},50}$ is the LC_{50} of a sample.

Genotoxicity Assay and Genotoxicity Equivalent

DNA double-strand breaks (DSBs) were characterized and quantified by the phosphorylated H2AX histone (pH2AX) as our previous study reported.[10] Cells were

seeded in a sterile 96-well plate (Corning 3599, U.S.A.) and incubated for 24 h, and then exposed to samples at different concentrations for 24 h. DMEM/F12 containing 0.5% DMSO was used as the negative control, while 4-nitroquinoline N-oxide (4-NQO) dissolved in DMEM/F12 containing 0.5% DMSO was used as the reference compound. After exposure for 24 h, cells were fixed with paraformaldehyde, permeated with Triton-100, and blocked with bovine serum albumin. Cells were incubated with the primary antibody phospho-histone H2AX and then stained with the second antibody Alexa Fluor® 647 conjugate together with Hoechst 33258. After the staining, images of the cells were obtained using an HCA system (ImageXpress® Micro, Molecular Devices, USA) with a 40× objective lens. The pH2AX foci were obtained from the CY5 channel and nucleus DNA was obtained from the DAPI channel. In each well of the plate, the number of pH2AX per cell was calculated by the total number of pH2AX foci over the number of nucleus. Each test was performed in 4-8 replicates.

The pH2AX induction rate of a sample was calculated as follows:

$$IR=\frac{(\text{pH2AX per cell})_S}{(\text{pH2AX per cell})_n}$$

where IR is the pH2AX induction rate for a sample or 4-NQO against the negative control. The $(\text{pH2AX per cell})_s$ is the number of pH2AX foci induced by the sample or 4-NQO. The $(\text{pH2AX per cell})_n$ is the number of pH2AX foci induced by the negative control.

The pH2AX foci induction ratios of samples at different concentration factors with cell viabilities >70% were used to obtain concentration–effect curves by regression analysis. The genotoxicity equivalent of a sample was calculated as follows:

$$\text{Genotoxicity equivalent}=\frac{(IR_{1.5})_{4\text{-NQO}}}{(IR_{1.5})_{\text{Sample}}}$$

where the unit of genotoxicity equivalent is µg–4-NQO/L, $(IR_{1.5})_{4\text{-NQO}}$ is the concentration of the 4-NQO solution that leads to the 1.5-fold induction ratio over the negative control, and $(IR_{1.5})_{\text{Sample}}$ is the concentration of a sample that leads to the 1.5

fold induction ratio over the negative control.

Measurement of Intracellular Reactive Oxygen Species

Reactive oxygen species (ROS) were measured using an intracellular total ROS activity assay kit (KA4075, Abnova, China). Cells were seeded into a sterile 96-well plate (3603 Corning, New York, U.S.A.) and incubated for 24 h, and then exposed to samples at different concentrations factors for 24 h. Then, the culture medium was removed, and 30 μL of cell permeating nonfluorescent ROS probe was added and incubated for 1 h. The cell nuclei were then stained with Hoechst 33258. Cell images were recorded using the HCA system, ROS were recorded using the tetramethylrhodamine isothiocyanate (TRITC) channel, and cell nuclei were recorded using the 4',6-diamidino-2-phenylindole dihydrochloride (DAPI) channel. MetaXpress software was used to analyze the fluorescence signal. The total fluorescence intensity was divided by the cell number to obtain the ROS level in each cell. Each test was performed in 4-8 replicates.

Measurement of 8-hydroxy-(deoxy)guanosine (8-OH(d)G)

Cells were seeded in a sterile 96-well plate (Corning 3599, U.S.A.) and incubated for 24 h, and then exposed to samples at different concentrations factors for 24 h. The cells were then fixed, permeated, and blocked using the same method as was used for the pH2AX assay. The cells were then treated with a primary antibody (DNA/RNA oxidative damage markers; Thermo Fisher Scientific) for 1 h. Thereafter, cells were stained with the secondary antibody (Alexa Fluor plus 555 (Thermo Fisher Scientific)) and Hoechst 33258 for 1 h. Images of the cells were then acquired using the HCA system using the TRITC channel for 8-OH(d)G and the DAPI channel for the nuclei. The 8-OH(d)G concentration in cells was defined as the total fluorescence intensity divided by the number of cells and was expressed as the fluorescence intensity per cell.

Each test was performed in 4-8 replicates.

Supporting Figures

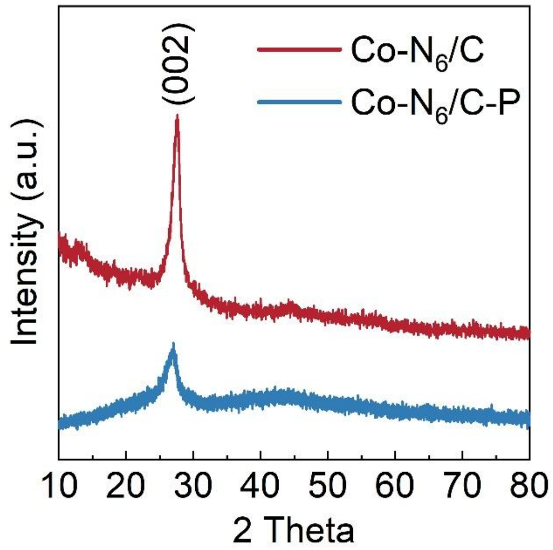

Figure S1. XRD patterns of $Co-N_6/C$ and $Co-N_6/C-P$.

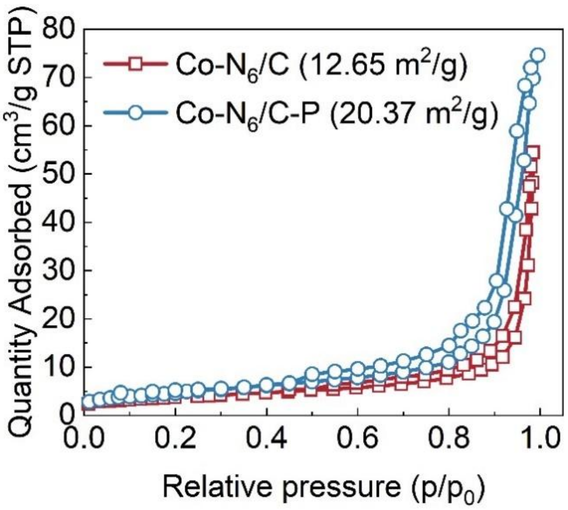

Figure S2. N_2 adsorption and desorption isotherms of Co-N_6/C and Co-N_6/C-P.

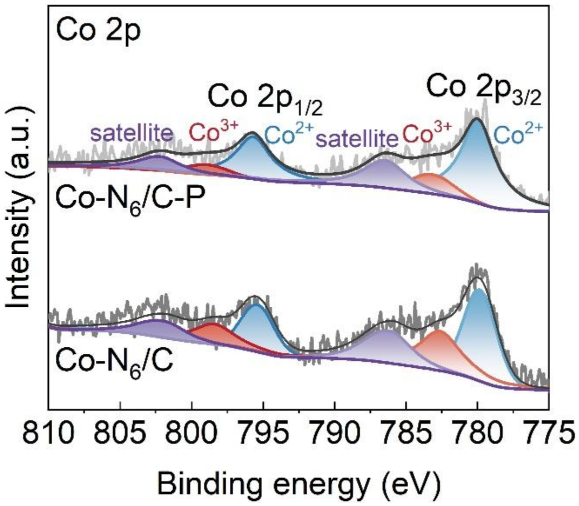

Figure S3. Co 2p XPS spectra of Co-N_6/C and Co-N_6/C-P.

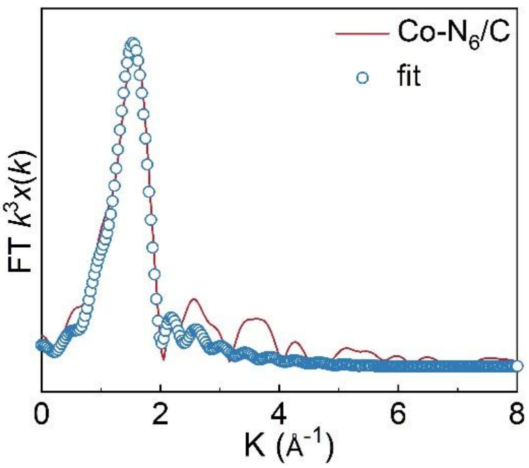

Figure S4. Co K-edge EXAFS fitting analyses for Co-N_6/C.

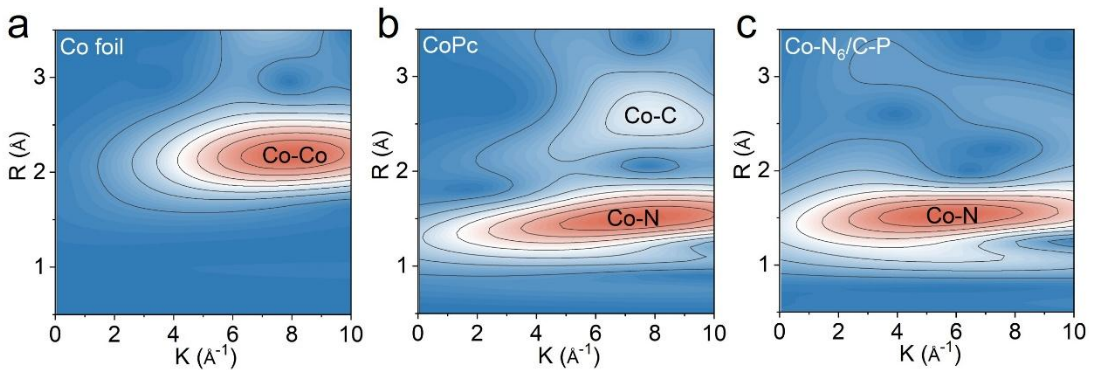

Figure S5. Co K-edge WT-EXAFS contour plots of Co foil, CoPc, and Co-N_6/C-P.

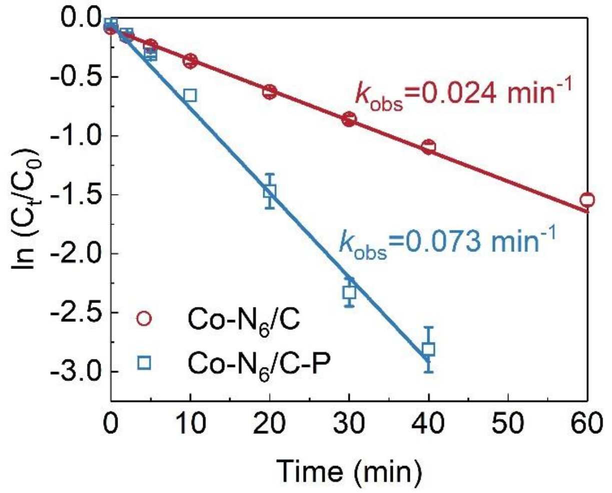

Figure S6. The pseudo first-order kinetic model fitting of APAP. Reaction condition: [catalyst] = 0.1 g/L, [PMS] = 0.1 mM, [APAP] = 2 mg/L, initial pH = 4.37.

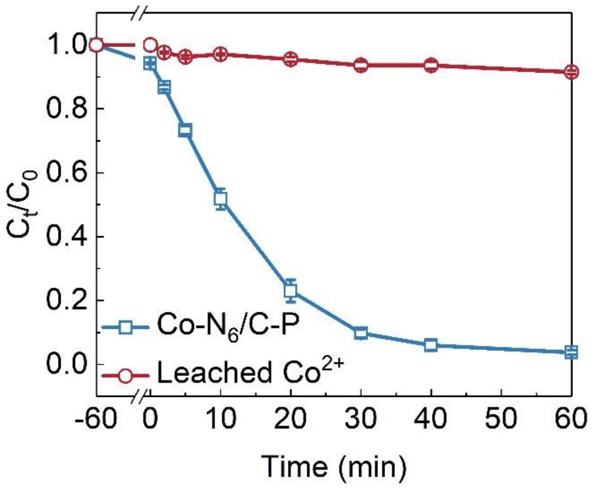

Figure S7. APAP degradation by Co-N_6/C-P and leached Co^{2+}. Reaction condition: [catalyst] = 0.1 g/L, [PMS] = 0.1 mM, [APAP] = 2 mg/L, initial pH = 4.37.

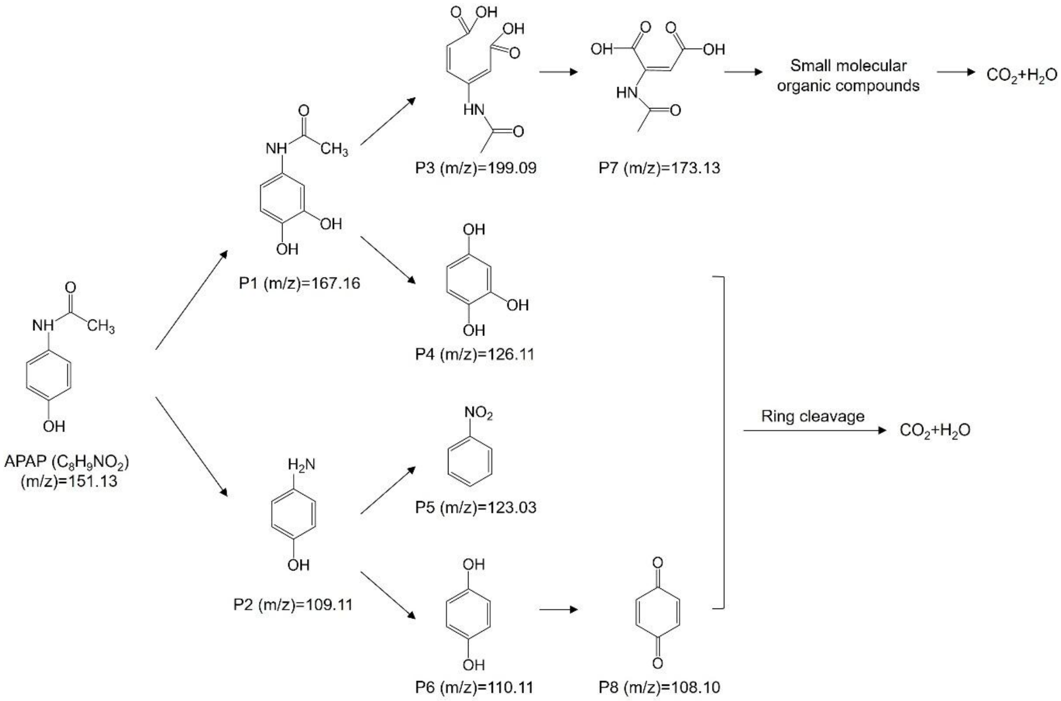

Figure S8. Proposed degradation pathway of APAP in Co–N_6/C–P/PMS system.

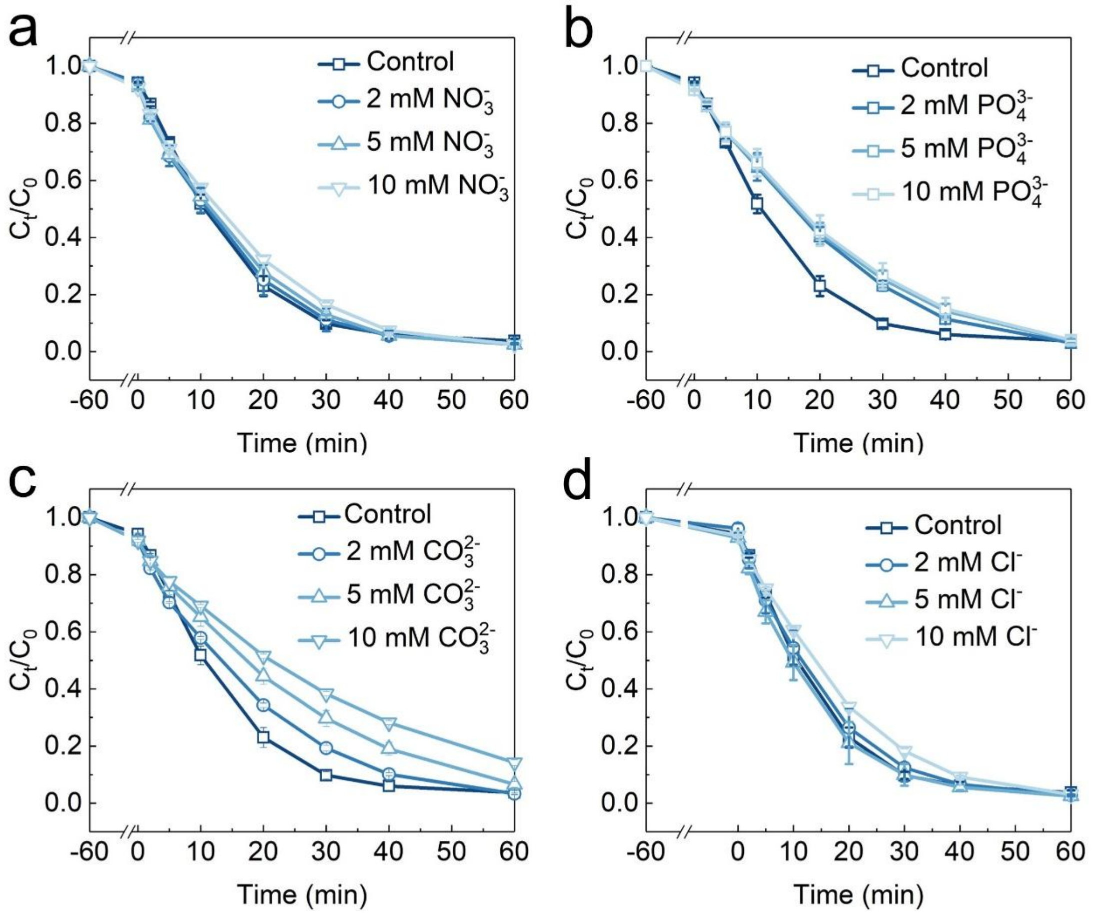

Figure S9. The effect of the co-existing anions on APAP degradation: (a) NO_3^-, (b) PO_4^{3-}, (c) CO_3^{2-}, and (d) Cl^-. Reaction condition: [catalyst] = 0.1 g/L, [PMS] = 0.1 mM, [APAP] = 2 mg/L, initial pH = 4.37.

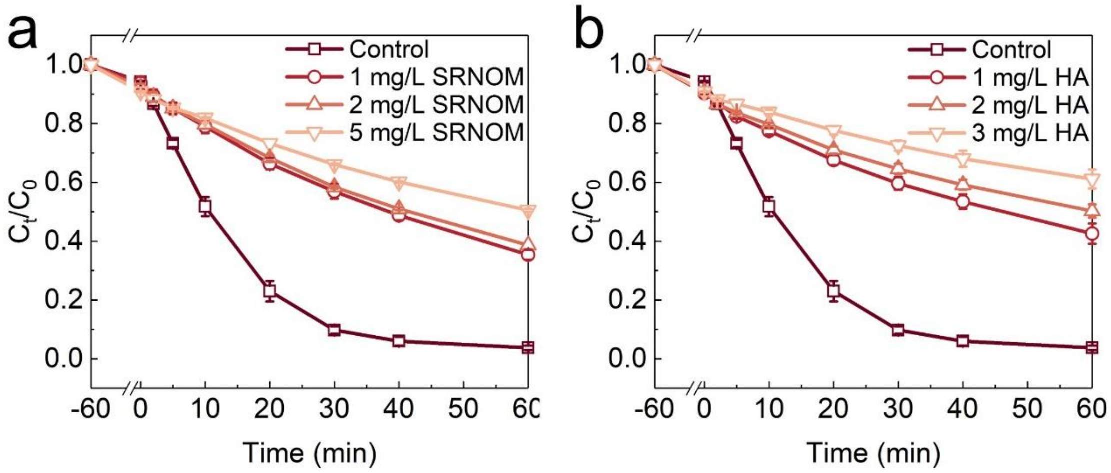

Figure S10. The effect of organic matter on APAP degradation: (a) natural organic matter from Suwannee River (SRNOM), and (b) humic acid (HA). Reaction condition: [catalyst] = 0.1 g/L, [PMS] = 0.1 mM, [APAP] = 2 mg/L, initial pH = 4.37.

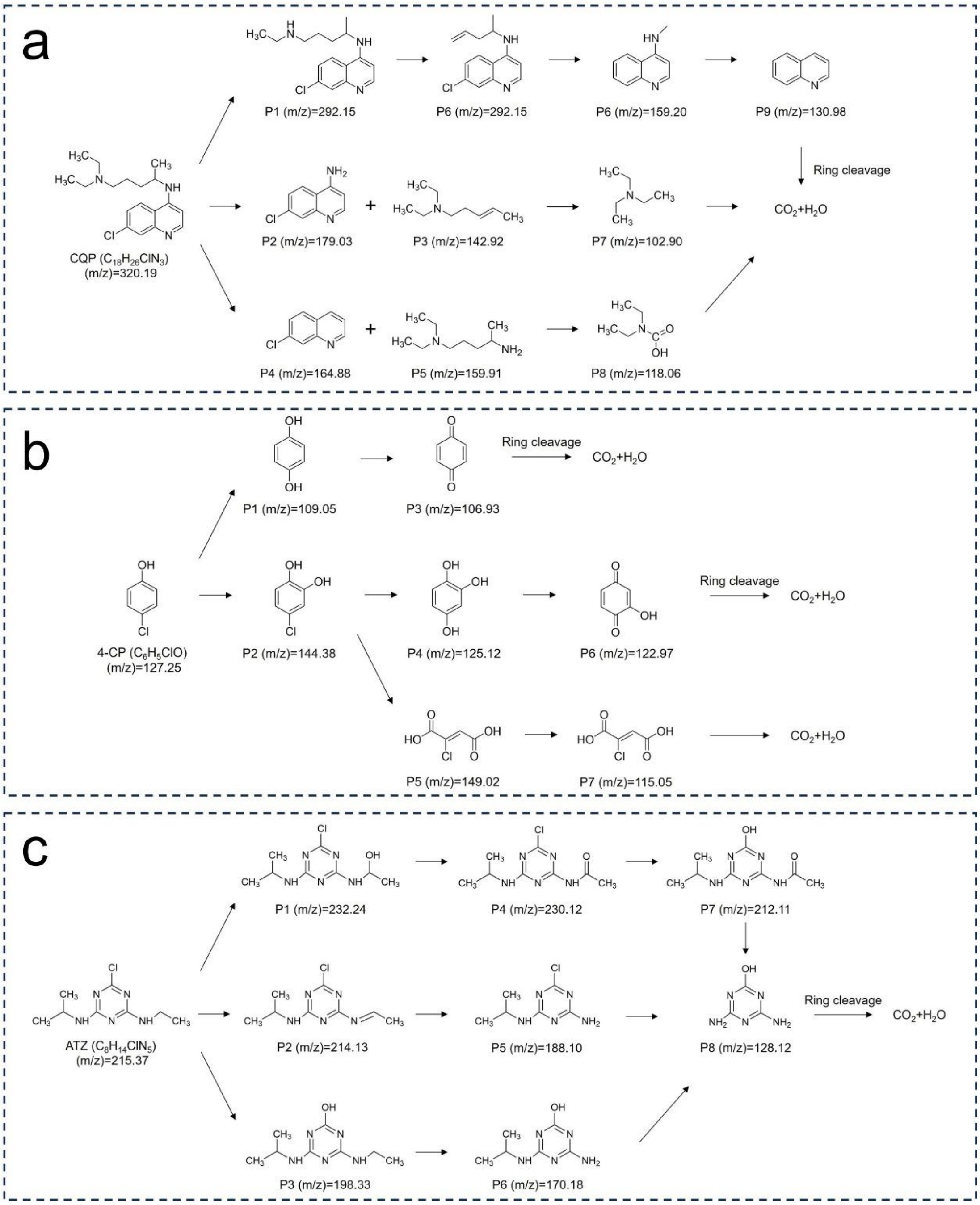

Figure S11. Proposed degradation pathway of (a) CQP, (b) 4-CP and (c) ATZ in Co–N_6/C–P/PMS system.

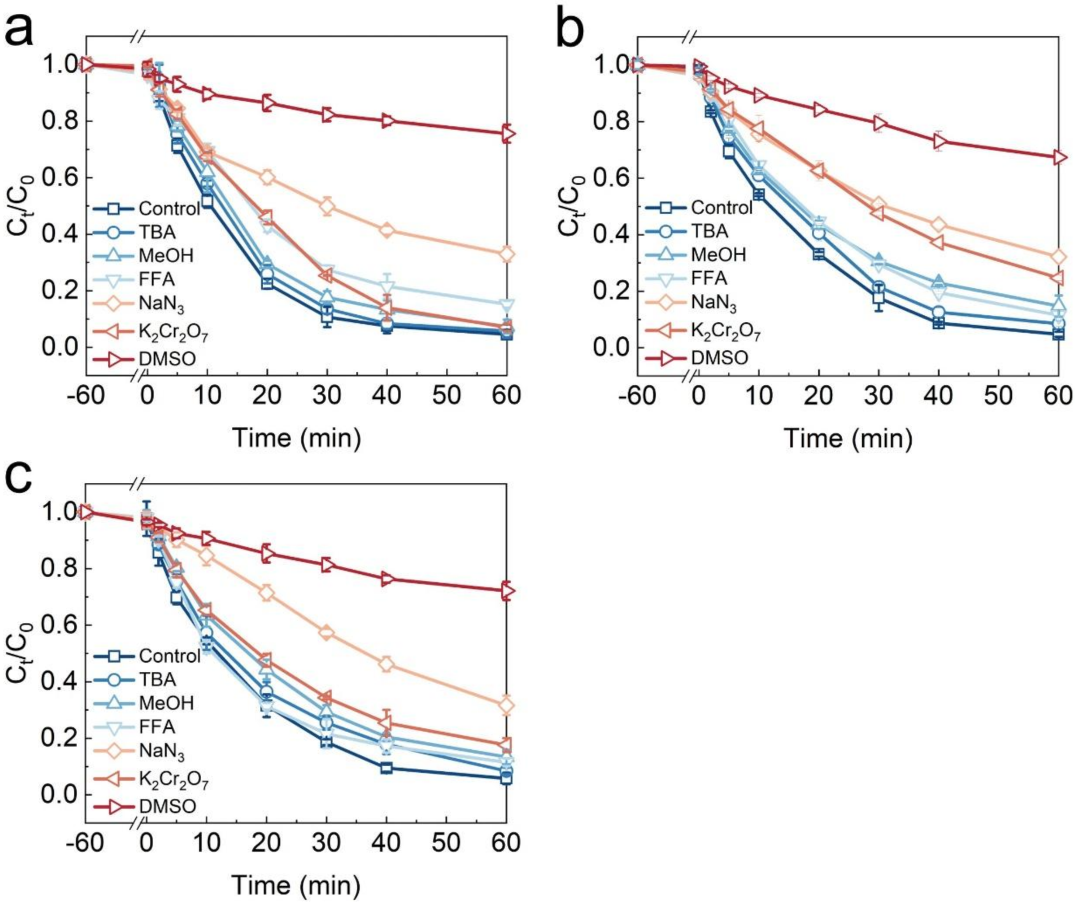

Figure S12. Quenching experiments of the (Co-N_6/C-P)/PMS system for the degradation of (a) CQP, (b) 4-CP, and (c) ATZ.

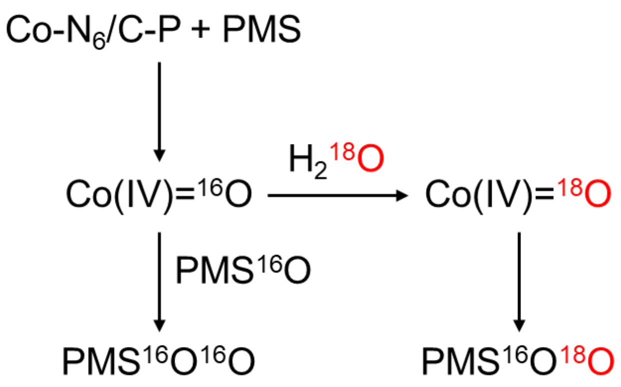

Figure S13. The schematic diagram of ^{18}O-isotope-labeled $PMSO_2$ production process.

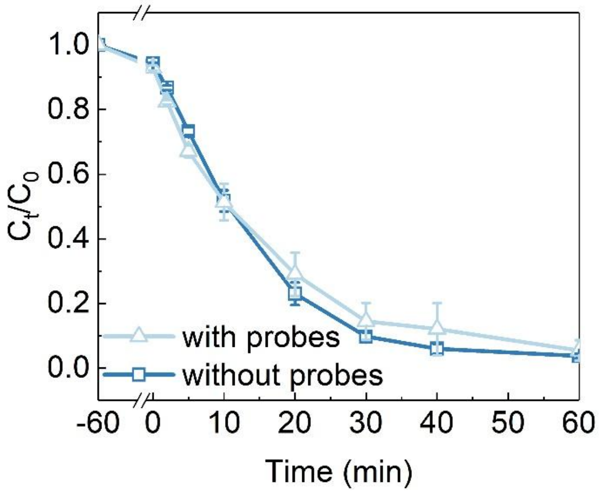

Figure S14. The degradation of APAP in the (Co-N_6/C-P)/PMS system with and without probes. Reaction condition: [catalyst] = 0.1 g/L, [PMS] = 0.1 mM, [APAP] = 2 mg/L, [BA]=[NB]=[FFA]= [*p*CBA]=0.2mg/L (if needed), [PMSO]= 1μM (if needed), initial pH = 4.37.

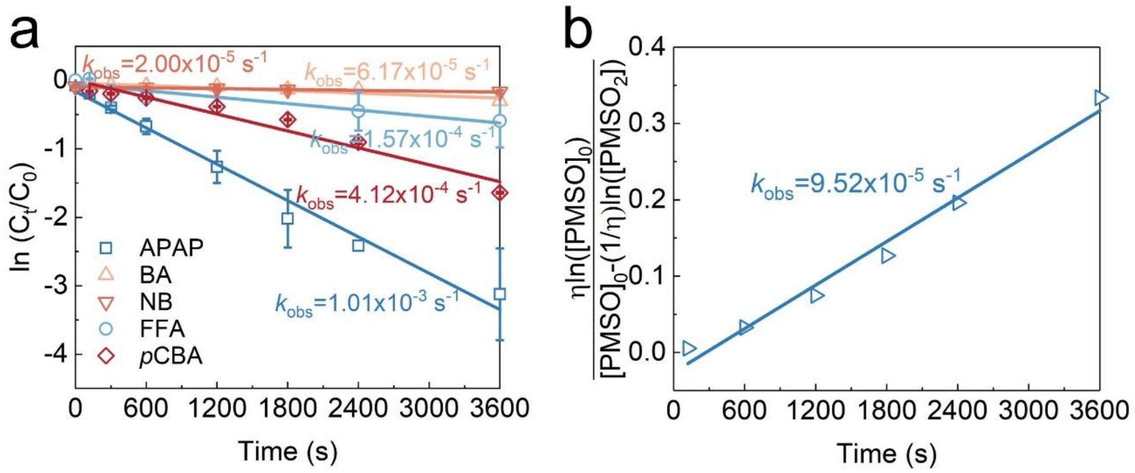

Figure S15. (a) Pseudo first-order kinetic model fitting of APAP and probes. (b) The pseudo-first-order reaction rate constants of $PMSO_2$. Reaction condition: [Reaction condition: [catalyst] = 0.1 g/L, [PMS] = 0.1 mM, [APAP] = 2 mg/L, [catalyst] = 0.1 g/L, [PMS] = 0.1 mM, [APAP] = 2 mg/L, [BA]=[NB]=[FFA]= [*p*CBA]=0.2mg/L (if needed), [PMSO]= 1μM (if needed), initial pH = 4.37.

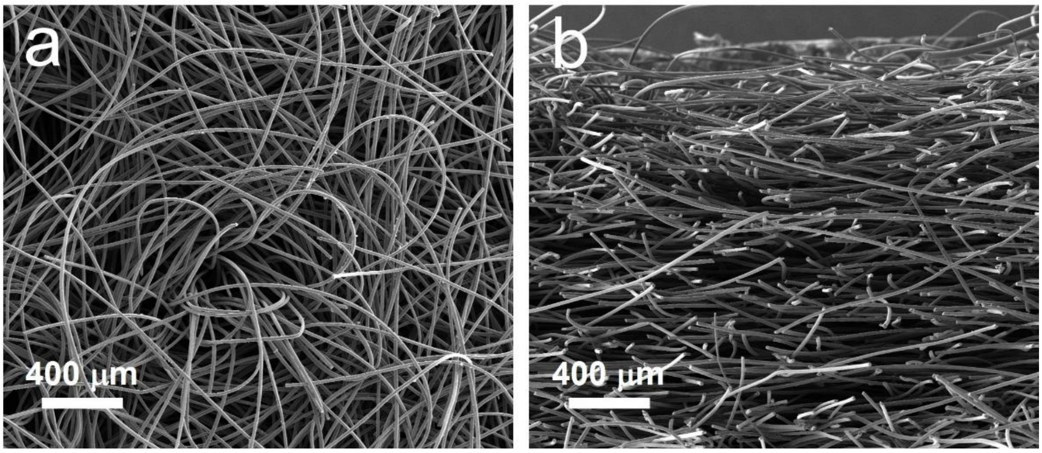

Figure S16. SEM images of Co-N_6/C-P-loaded carbon felt: (a) front side, and (b) cross section.

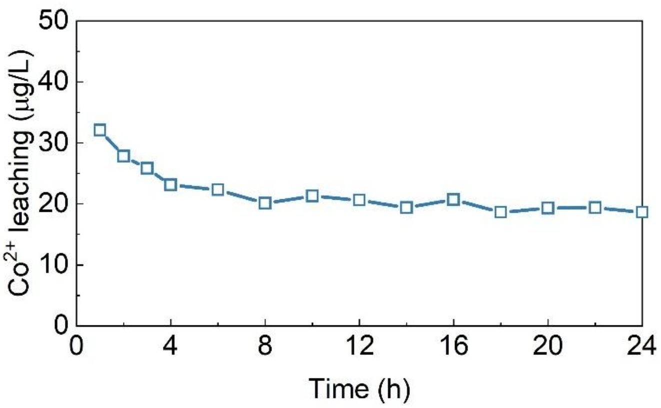

Figure S17. Leaching of Co ions in the carbon felt–Co–N_6/C–P/PMS continuous-flow system.

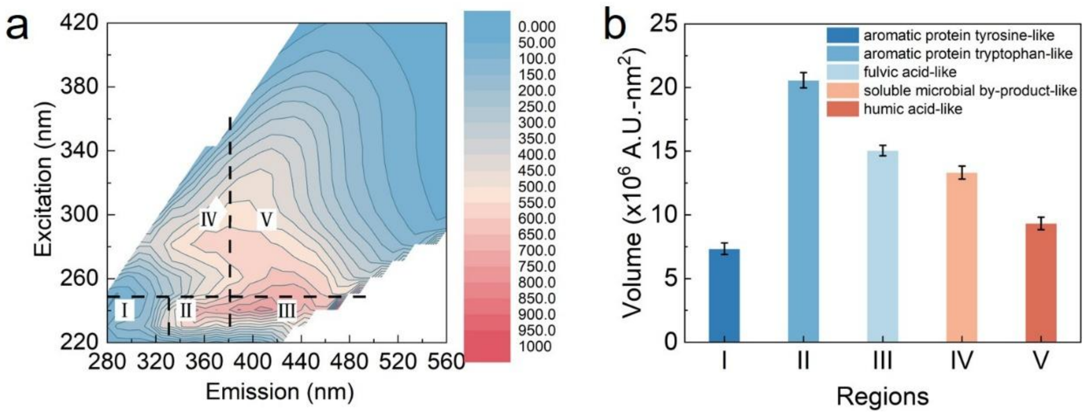

Figure S18. (a) Three-dimensional excitation-emission matrix (3D-EEM) and (b) fluorescence intensity of secondary effluent.

Supporting Tables

Table S1. The mass ratios of Co in Co-N_6/C and Co-N_6/C-P.

Catalysts	Co content (wt.%)
Co-N_6/C	3.54
Co-N_6/C-P	3.92

Table S2. EXAFS fitting parameters at the Co K-edge for Co-N_6/C and Co-N_6/C-P (S_0^2=0.71).

	shell	CN	R(Å)	σ^2	ΔE_0	R factor
Co foil	Co-Co	12	2.49±0.01	0.0062	7.8±0.3	0.0012
CoPc	Co-N	4.0±0.1	1.97±0.02	0.0021	6.5±1.8	0.0011
Co-N_6/C	Co-N	5.9±0.4	2.01±0.01	0.0089	-2.2±0.7	0.0032
Co-N_6/C-P	Co-N	5.7±0.2	2.02±0.01	0.0112	-2.4±1.1	0.0049

[a]*N*: coordination numbers; [b]*R*: bond distance; [c]σ^2: Debye-Waller factors; [d] ΔE_0: the inner potential correction. *R* factor: goodness of fit.

Table S3. Comparison of micropollutants degradation of by (Co-N_6/C-P)/PMS and other single-atom catalyst/PMS systems.

Catalyst	Catalyst dosage (g/L)	PMS dosage (g/L)	Micropollutant dosage (mg/L)	k_{obs} (min^{-1})	k_N ($min^{-1}g^{-2}$)	Ref.
Fe-SAC (0.2)	0.2	0.4	bisphenol A (25)	0.104	1.30	[11]
Fe_1-CN	0.5	6.15	4-Chlorophenol (12.86)	0.55	0.18	[12]
Fe-SA/PHCNS	0.1	0.2	Acetaminophen (10)	0.437	21.85	[13]
SA-Fe-g-C_3N_4	0.1	0.154	Tetracycline (10)	0.044	2.86	[14]
Cu-N_4/C	0.1	0.2	Bisphenol A (20)	0.102	5.10	[15]
Cu-N_4/C-B	0.1	0.2	Bisphenol A (20)	0.56	28.00	[15]
Mn-CN	0.05	0.8	Acetaminophen (20)	0.103	2.58	[16]
Co-N_4	0.1	0.4	Ciprofloxacin (5)	0.206	5.15	[17]
Co-N_3O_1	0.1	0.4	Ciprofloxacin (5)	0.287	7.18	[17]
Co-TPML	0.2	0.615	Bisphenol A (11.4)	2.6	21.14	[18]
Co-N_6/C	0.1	0.02	Acetaminophen (2)	0.024	12.00	This work
Co-N_6/C-P	0.1	0.02	Acetaminophen (2)	0.073	36.50	This work

Table S4. Degradation intermediates of APAP.

Product	Formula	m/z	Proposed structure
APAP	$C_8H_9NO_2$	151.13	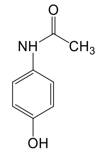
P1	$C_8H_9NO_3$	167.16	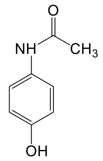
P2	C_6H_7NO	109.11	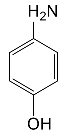
P3	$C_8H_9NO_5$	199.09	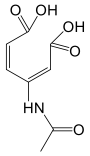
P4	$C_6H_6O_3$	126.11	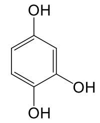
P5	$C_6H_5NO_2$	123.03	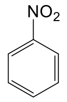
P6	$C_6H_6O_2$	110.11	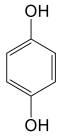
P7	$C_6H_8NO_5$	173.13	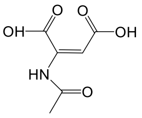
P8	$C_6H_4O_2$	108.10	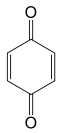

Table S5. Second-order rate constants for reactions between quenchers with various reactive species (M^{-1} s^{-1}).

Quencher	Quenched species	$k_{\bullet OH}$	$k_{SO4\bullet-}$	k_{1O2}	$k_{O2\bullet-}$	$k_{Co(IV)=O}$	Ref.
TBA	·OH	6×10^{8}	4×10^{5}	1.8×10^{3}	N/A	$<1.0\times10^{2}$	[19-21]
MeOH	·OH, $SO_4^{\bullet-}$	9.7×10^{8}	1.1×10^{7}	3.89×10^{3}	N/A	6.1×10^{3}	[19-21]
FFA	·OH, $SO_4^{\bullet-}$, $^1O_2$	1.5×10^{10}	1.3×10^{10}	1.2×10^{8}	3.5×10^{3}	N/A	[19, 20]
NaN_3	·OH, $SO_4^{\bullet-}$, $^1O_2$, $O_2^{\cdot-}$	1.2×10^{9}	2.52×10^{9}	1.2×10^{10}	6.4×10^{8}	N/A	[19, 20, 22]
DMSO	·OH, $SO_4^{\bullet-}$, Co(IV)=O	4.5×10^{9}	3×10^{9}	5.2×10^{4}	N/A	2.4×10^{6}	[21, 23-25]

Table S6. The second-order reaction rate constants between quenchers with various reactive species (M^{-1} s^{-1}).

Quencher	$k_{•OH}$	$k_{SO4•-}$	k_{1O2}	$k_{O2•-}$	$k_{Co(IV)=O}$	Ref.
BA	1.2×10^9	5.9×10^9	N/A	N/A	N/A	[26-28]
NB	3.9×10^9	$<10^6$	N/A	N/A	N/A	[27-29]
FFA	1.5×10^{10}	1.3×10^{10}	1.2×10^8	3.5×10^3	N/A	[19, 20]
*p*CBA	5.0×10^9	3.6×10^8	1.4×10^7	8.6×10^7	N/A	[19, 26, 30]
PMSO	3.61×10^9	3.17×10^8	N/A	N/A	2×10^6	[21, 31]
APAP	1.7×10^9	1.3×10^9	3.4×10^5	2.7×10^3	9.59×10^6	[28, 32, 33]

Table S7. Spectral region and the corresponding substance types used in 3D-EEM of Fig. S18.

Category	Wavelength range (nm)		Type
	Excitation	Emission	
I	220–250	280–330	Aromatic protein tyrosine-like
II	220–250	330–380	Aromatic protein tryptophan-like
III	220–250	380–480	Fulvic acid-like
IV	250–360	280–380	Soluble microbial by-product-like
V	250–420	380–520	Humic acid-like

Supplementary References

[1] C. Liang, C.F. Huang, N. Mohanty, R.M. Kurakalva, A rapid spectrophotometric determination of persulfate anion in ISCO, Chemosphere, 2008, 73(9), 1540-1543.

[2] Q.Y. Wu, Z.W. Yang, Z.W. Wang, W.L. Wang, Oxygen doping of cobalt-single-atom coordination enhances peroxymonosulfate activation and high-valent cobalt-oxo species formation, Proc. Natl. Acad. Sci. 2023, 120(16), e2219923120.

[3] G. Kresse, J. Furthmüller, Efficiency of ab-initio total energy calculations for metals and semiconductors using a plane-wave basis set, Comput. Mater. Sci, 1996, 6(1), 15-50.

[4] J.P. Perdew, K. Burke, M. Ernzerhof, Generalized gradient approximation made simple, Phys. Rev. Lett. 1996, 77, 3865.

[5] H.J. Monkhorst, J.D. Pack, Special points for Brillouin-zone integrations, Phys. Rev. B: Condens. Matter Mater. Phys. 1976, 13(12), 5188-5192.

[6] P.E. Blchl, Projector augmented-wave method, Phys. Rev. B: Condens. Matter Mater. Phys. 1995, 50(24), 17953-17979.

[7] G. Henkelman, B.P. Uberuaga, H. Jónsson, A climbing image nudged elastic band method for finding saddle points and minimum energy paths, J. Chem. Phys. 2000, 113(22), 9901-9904.

[8] A.A. Peterson, F. Abild-Pedersen, F. Studt, J. Rossmeisl, J.K. Nørskov, How copper catalyzes the electroreduction of carbon dioxide into hydrocarbon fuels, Energy Environ. Sci. 2010, 3, 1311-1315.

[9] Q.Y. Wu, L.L. Yang, X.Y. Zhang, W.L. Wang, Y. Lu, Y. Du, Y. Lu, H.Y. Hu, Ammonia-mediated bromate inhibition during ozonation promotes the toxicity due to organic byproduct transformation, Environ. Sci. Technol. 2020, 54(14), 8926-8937.

[10] Y. Du, W.L. Wang, Z.-W. Wang, C.J. Yuan, M.Q. Ye, Q.Y. Wu, Overlooked cytotoxicity and genotoxicity to mammalian cells caused by the oxidant peroxymonosulfate during wastewater treatment compared with the sulfate radical-based ultraviolet/peroxymonosulfate process, Environ. Sci. Technol. 2023, 57(8), 3311-3322.

[11] Y. Gao, T. Wu, C. Yang, C. Ma, Z. Zhao, Z. Wu, S. Cao, W. Geng, Y. Wang, Y. Yao, Y. Zhang, C. Cheng, Activity trends and mechanisms in peroxymonosulfate-assisted catalytic production of singlet oxygen over atomic metal-N-C catalysts, Angew. Chem. Int. Ed. 2021, 60(41), 22513-22521.

[12] L.S. Zhang, X.H. Jiang, Z.A. Zhong, L. Tian, Q. Sun, Y.T. Cui, X. Lu, J.P. Zou, S.L. Luo, Carbon nitride supported high-loading Fe single-atom catalyst for activation of peroxymonosulfate to generate $^1O_2$ with 100 % selectivity, Angew. Chem. Int. Ed. 2021, 60(40), 21751-21755.

[13] Z. Wang, W. Wang, J. Wang, Y. Yuan, Q. Wu, H. Hu, High-valent iron-oxo species mediated cyclic oxidation through single-atom Fe-N_6 sites with high peroxymonosulfate utilization rate, Appl. Catal., B, 2022, 305, 121049.

[14] X. Peng, J. Wu, Z. Zhao, X. Wang, H. Dai, L. Xu, G. Xu, Y. Jian, F. Hu, Activation

of peroxymonosulfate by single-atom Fe-g-C_3N_4 catalysts for high efficiency degradation of tetracycline via nonradical pathways: Role of high-valent iron-oxo species and Fe–N_x sites, Chem. Eng. J. 2022, 427, 130803.

[15] X. Zhou, M.K. Ke, G.X. Huang, C. Chen, W. Chen, K. Liang, Y. Qu, J. Yang, Y. Wang, F. Li, H.Q. Yu, Y. Wu, Identification of Fenton-like active Cu sites by heteroatom modulation of electronic density, Proc. Natl. Acad. Sci. 2022, 119(8).

[16] X. Li, X. Huang, S. Xi, S. Miao, J. Ding, W. Cai, S. Liu, X. Yang, H. Yang, J. Gao, J. Wang, Y. Huang, T. Zhang, B. Liu, Single cobalt atoms anchored on porous N-doped graphene with dual reaction sites for efficient Fenton-like catalysis, J. Am. Chem. Soc. 2018, 140(39), 12469-12475.

[17] Z. Wang, E. Almatrafi, H. Wang, H. Qin, W. Wang, L. Du, S. Chen, G. Zeng, P. Xu, Cobalt single atoms anchored on oxygen-doped tubular carbon nitride for efficient peroxymonosulfate activation: simultaneous coordination structure and morphology modulation, Angew. Chem. Int. Ed. 2022, 61(29), e202202338.

[18] C. Chu, J. Yang, X. Zhou, D. Huang, H. Qi, S. Weon, J. Li, M. Elimelech, A. Wang, J.-H. Kim, Cobalt single atoms on tetrapyridomacrocyclic support for efficient peroxymonosulfate activation, Environ. Sci. Technol. 2021, 55(2), 1242-1250.

[19] Y. Guo, J. Long, J. Huang, G. Yu, Y. Wang, Can the commonly used quenching method really evaluate the role of reactive oxygen species in pollutant abatement during catalytic ozonation?, Water Res. 2022, 215, 118275.

[20] L. Gao, Y. Guo, J. Zhan, G. Yu, Y. Wang, Assessment of the validity of the quenching method for evaluating the role of reactive species in pollutant abatement during the persulfate-based process, Water Res. 2022, 221, 118730.

[21] Y. Zong, X. Guan, J. Xu, Y. Feng, Y. Mao, L. Xu, H. Chu, D. Wu, Unraveling the Overlooked Involvement of High-Valent Cobalt-Oxo Species Generated from the Cobalt(II)-Activated Peroxymonosulfate Process, Environ. Sci. Technol. 2020, 54(24), 16231-16239.

[22] A. Khan, K. Zhang, A. Taraqqi-A-Kamal, X. Wang, Y. Chen, Y. Zhang, Degradation of antibiotics in aqueous media using manganese nanocatalyst-activated peroxymonosulfate, J. Colloid Interface Sci. 2021, 599, 805-818.

[23] L. Zhu, J.M. Nicovich, P.H. Wine, Temperature-dependent kinetics studies of aqueous phase reactions of SO_4^- radicals with dimethylsulfoxide, dimethylsulfone, and methanesulfonate, J. Photoch. Photobio. A, 2003, 157(2), 311-319.

[24] H. Bardouki, M.B. da Rosa, N. Mihalopoulos, W.U. Palm, C. Zetzsch, Kinetics and mechanism of the oxidation of dimethylsulfoxide (DMSO) and methanesulfinate (MSI^-) by OH radicals in aqueous medium, Atmos. Environ. 2002, 36(29), 4627-4634.

[25] I. Kruk, H.Y. Aboul-Enein, T. Michalska, K. Lichszteld, K. Kubasik-Kladna, S. Olgen, In vitro scavenging activity for reactive oxygen species by N-substituted indole-2-carboxylic acid esters, Luminescence, 2007, 22(4), 379-86.

[26] B. Liu, W. Guo, H. Wang, S. Zheng, Q. Si, Q. Zhao, H. Luo, N. Ren, Peroxymonosulfate activation by cobalt(II) for degradation of organic contaminants via high-valent cobalt-oxo and radical species, J. Hazard. Mater. 2021, 416, 125679.

[27] S.-Y. Liou, M.C. Dodd, Evaluation of hydroxyl radical and reactive chlorine species generation from the superoxide/hypochlorous acid reaction as the basis for a novel advanced oxidation process, Water Res. 2021, 200, 117142.
[28] Z. Wang, W. Wang, J. Wang, Y. Yuan, Q. Wu, H. Hu, High-valent iron-oxo species mediated cyclic oxidation through single-atom Fe-N_6 sites with high peroxymonosulfate utilization rate, Appl. Catal. B, 2022, 305.
[29] Z.W. Yang, W.L. Wang, M.Y. Lee, Q.Y. Wu, Y.T. Guan, Synergistic effects of ozone/peroxymonosulfate for isothiazolinone biocides degradation: Kinetics, synergistic performance and influencing factors, Environ. Pollut. 2022, 294, 118626.
[30] J. Cong, G. Wen, T. Huang, L. Deng, J. Ma, Study on enhanced ozonation degradation of para-chlorobenzoic acid by peroxymonosulfate in aqueous solution, Chem. Eng. J. 2015, 264, 399-403.
[31] B. Liu, W. Guo, H. Wang, S. Zheng, Q. Si, Q. Zhao, H. Luo, N. Ren, Peroxymonosulfate activation by cobalt(II) for degradation of organic contaminants via high-valent cobalt-oxo and radical species, J. Hazard. Mater. 2021, 416, 125679.
[32] Y. Li, Y. Pan, L. Lian, S. Yan, W. Song, X. Yang, Photosensitized degradation of acetaminophen in natural organic matter solutions: The role of triplet states and oxygen, Water Res. 2017, 109, 266-273.
[33] Y. Yuan, D. Zhao, J. Li, F. Wu, M. Brigante, G. Mailhot, Rapid oxidation of paracetamol by Cobalt(II) catalyzed sulfite at alkaline pH, Catal. Today, 2018, 313, 155-160.
